# Integrated omics of *Saccharomyces cerevisiae* CENPK2-1C reveals pleiotropic drug resistance and lipidomic adaptations to cannabidiol

**DOI:** 10.1038/s41540-024-00382-0

**Published:** 2024-05-31

**Authors:** Erin Noel Jordan, Ramin Shirali Hossein Zade, Stephanie Pillay, Paul van Lent, Thomas Abeel, Oliver Kayser

**Affiliations:** 1 Technical Biochemistry, TU Dortmund University, Emil-Figge-Straße 66, 44227 Dortmund, Germany; 2 Delft Bioinformatics Lab, Delft University of Technology Van Mourik, Broekmanweg 6, 2628 XE Delft, The Netherlands; 3 Department of Biomedical Data Sciences, Leiden University Medical Center, Leiden, The Netherlands; 4 Leiden Center for Computational Oncology, Leiden University Medical Center, Leiden, The Netherlands; 5 Infectious Disease and Microbiome Program, Broad Institute of MIT and Harvard, 415 Main Street, Cambridge, MA 02142 USA

**Keywords:** Metabolic engineering, Metabolic engineering

## Abstract

Yeast metabolism can be engineered to produce xenobiotic compounds, such as cannabinoids, the principal isoprenoids of the plant *Cannabis sativa*, through heterologous metabolic pathways. However, yeast cell factories continue to have low cannabinoid production. This study employed an integrated omics approach to investigate the physiological effects of cannabidiol on *S. cerevisiae* CENPK2-1C yeast cultures. We treated the experimental group with 0.5 mM CBD and monitored CENPK2-1C cultures. We observed a latent-stationary phase post-diauxic shift in the experimental group and harvested samples in the inflection point of this growth phase for transcriptomic and metabolomic analysis. We compared the transcriptomes of the CBD-treated yeast and the positive control, identifying eight significantly overexpressed genes with a log fold change of at least 1.5 and a significant adjusted p-value. Three notable genes were *PDR5* (an ABC-steroid and cation transporter), *CIS1*, and *YGR035C*. These genes are all regulated by pleiotropic drug resistance linked promoters. Knockout and rescue of *PDR5* showed that it is a causal factor in the post-diauxic shift phenotype. Metabolomic analysis revealed 48 significant spectra associated with CBD-fed cell pellets, 20 of which were identifiable as non-CBD compounds, including fatty acids, glycerophospholipids, and phosphate-salvage indicators. Our results suggest that mitochondrial regulation and lipidomic remodeling play a role in yeast’s response to CBD, which are employed in tandem with pleiotropic drug resistance (PDR). We conclude that bioengineers should account for off-target product C-flux, energy use from ABC-transport, and post-stationary phase cell growth when developing cannabinoid-biosynthetic yeast strains.

## Introduction

Cannabinoids are naturally occurring compounds synthesized through secondary metabolism. Although initially linked only to *Cannabis sativa* by definition, researchers have discovered cannabinoids in several plant species, such as *Helichrysum umbraculigerum*^1^*, Radula marginata*^2^, and other species^3,4^, prompting the revision of their definition to include an isoprenyl moiety oriented towards a para-structure via a resorcinyl core. To produce cannabinoids, a transferase facilitates the prenylation of polyketide-synthase end-products, such as olivetolic acid, with geranylpyrophosphate (GPP) from the *in-planta* pathway known as the methylerythritol phosphate pathway (MEP). The scientific community has shown interest in cannabinoids in recent years due to their therapeutic potential and structural diversity, which coincides with an increase in cannabis availability through legal procurement in many countries across the globe.

Researchers wishing to study cannabinoids have traditionally focused on plant cultivation, however, *in-planta* enzyme screening poses challenges including lengthy growth cycles, limited emulation of crucial biotic and abiotic factors necessary for cannabinoid production, and high cultivation costs. Scientists can employ heterologous microbial engineering to overcome these limitations. Moreover, heterologous cloning methods offer unique insights into pathway deduction because of the ability to screen individual enzymes or combinations of enzymes that can easily be screened for metabolic activity. Cannabinoid research has therefore pivoted towards bioengineering yeast and other microbial organisms to serve as microbial cell factories. In general, this also boasts crucial advantages such as commercial benefit and scalability^5^, reduced environmental harm, and consumer safety through reduced risk of mycotoxins or heavy metals to consumers^6^.

**Fig. 1:**
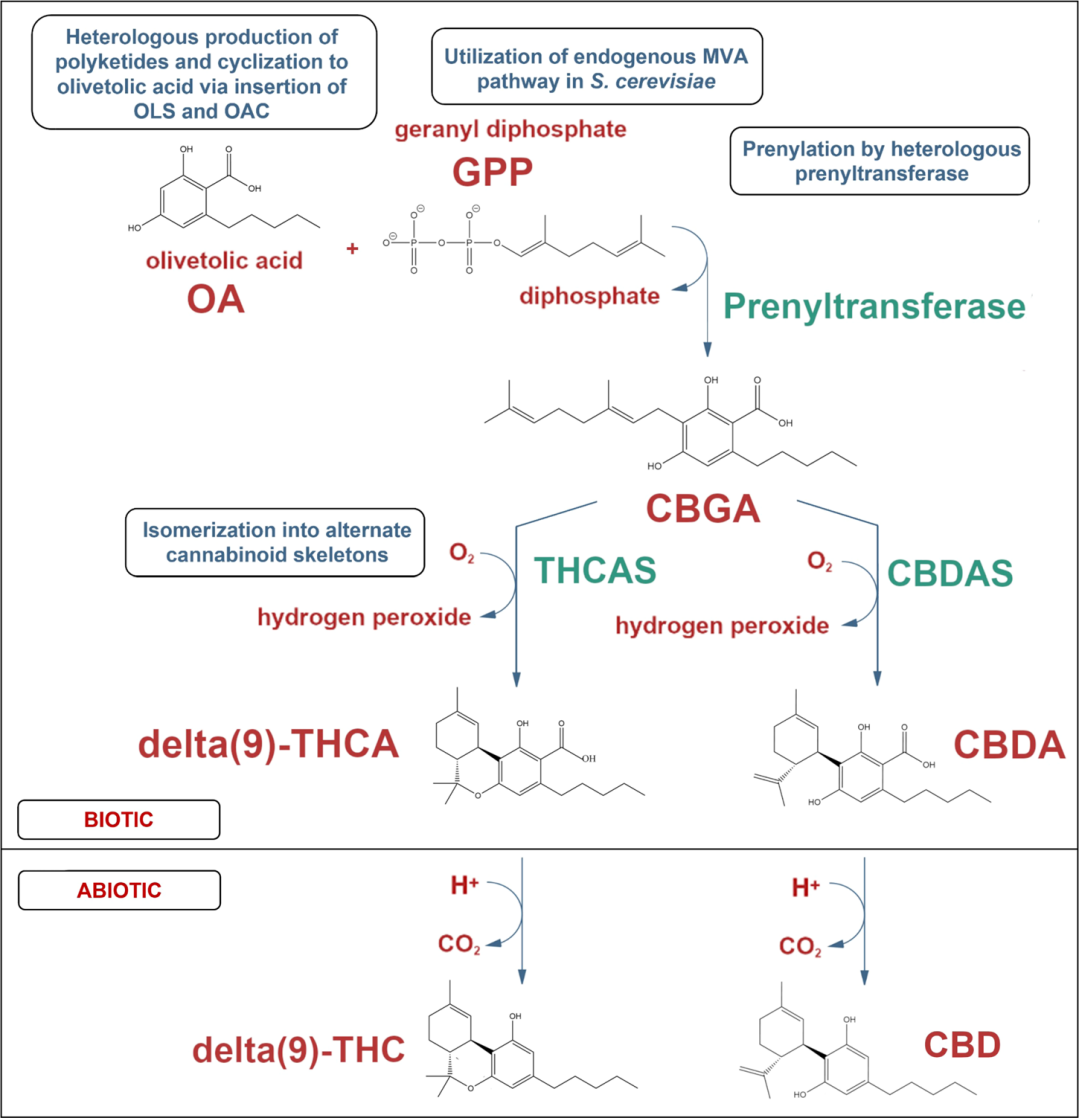
General overview of the biosynthesis of cannabinoids. In a yeast cell factory, polyketide synthesis is heterologously inserted and the naturally occurring fatty acid metabolism of yeast is exploited to produce olivetolic acid (OA). The endogenous mevalonate pathway of yeast is then manipulated to overproduce geranyl diphosphate (GPP). GPP and OA are then prenylated together into cannabigerolic acid (CBGA) by a heterologous prenyltransferase. Last, heterologous isomerases are employed to produce alternate cannabinoid skeletal arrangements such as Δ9-THCA and CBDA, where these could then abiotically be decarboxylated into Δ9-THC and CBD.

*Saccharomyces cerevisiae* is an appealing eukaryote for developing yeast cell factories (YCFs) that produce cannabinoids^7–9^. Researchers have already established cannabinoid biosynthetic YCF’s^10^, among many other YCFs to advance the production of pharmaceuticals, vitamins, natural flavors, and terpenoids. Yeast is known for efficient expression and regulation of heterologous enzymes and, further, is considered a generally-recognized-as-safe organism^11^. In cannabinoid engineering, scientists can use yeast’s natural fatty acid metabolism to produce polyketides using heterologous polyketide synthases. Manipulation of the endogenous mevalonate (MVA) pathway (a GPP-producing pathway similar to MEP) and introduction of a heterologous prenyltransferase to create the cannabinoid cannabigerolic acid (CBGA) during cannabinoid biosynthesis, completes the cannabinoid pathway. Figure 1 depicts the pathways to include in a cannabinoid biosynthetic YCF. However, producing yeast strains and scaling cannabinoid production to a commercially viable quantity has been a challenge despite efforts made over the past decade. This is due to low cannabinoid titers, as noted by Luo et al. and ref. ^12^. Thomas et al. ^13^ proposed an *in-silico* pathway model, which integrated enzyme kinetic data, indicating that yeast could synthesize cannabinoids up to a theoretical concentration of 300 mg/L during a 40-h batch fermentation. While the work of Zhang et al. has recently made substantial progress towards realizing this theoretical maximum, their titer of 510 mg/L in 120 h still leaves potential room for improvement.

However, the model of Thomas ignored the possibility of metabolic disruptions caused by the accumulation of cannabinoids. Research indicates that cannabinoids, specifically CBD and THC, affect cellular physiology through mechanisms other than CB1 and CB2 interactions. These mechanisms include perturbation of mitochondrial^14–17^ and glucose metabolism^18^, as well as inhibition of α-glucosidases^19,20^. Furthermore, cannabidiol has been implicated in disrupting ion homeostasis and membrane integrity^18^. Although yeast lacks mammalian CB1 or CB2 homologs, it still depends on oxidative respiration through mitochondria, employs α-glucosidases in glucose metabolism, and establishes necessary membrane potentials with ion transport, rendering them vulnerable to physiological effects caused by cannabinoids. Yeast may adapt to xenobiotic accumulation from heterologous metabolic processes by modifying their plasma membrane^21,22^, which could disrupt lipid metabolism. This could include changes to the lipid profile of the plasma membrane to adjust its viscosity or porosity, or adjustments to the length of the lipids to thicken the membrane. Dynamic alterations in lipid metabolism impose energetic expenses on the cell and may also redirect the supply of essential precursors, such as acetyl-CoA and malonyl-CoA, from the cannabinoid pathway. Additionally, modifications to steroid metabolism may directly impede the function of enzymes in the MVA pathway and result in feedback inhibition of cannabinoid synthesis^23,24^. Further, ref. ^25^ discovered that JWH-018, a synthetic cannabimimetic molecule, stimulated glycolytic metabolism and inhibited pentose phosphate pathway activity, resulting in increased cellular growth of yeast. Finally, yeast cells exhibit a pleiotropic drug resistance response to xenobiotic molecules by overexpressing ABC transporters to eliminate exogenous compounds, such as anti-cancer or anti-malarial drugs^26–28^. ABC transporters impose energetic costs on the cell and incur metabolic expenses. It remains unclear whether cannabinoid production by yeasts is associated with pleiotropic drug resistance (PDR) and, if so, the threshold concentration at which this effect would manifest has not been determined. It would be interesting to bioengineers if a pleiotropic drug resistance response is activated at titers lower than the theoretical yield of cannabinoids in YCF’s. For all the reasons stated above, assessing the cellular effects of cannabinoid accumulation is crucial, and a comprehensive approach to understanding the underlying yeast physiology in response to cannabinoids should be taken.

We therefore investigated the growth patterns of yeast cultures treated with cannabinoids and analyzed the resulting transcriptomic and metabolic changes. Our objective was to understand the influence of cannabinoids on yeast metabolism, promote cannabinoid production, and potentially reveal wider implications for cannabinoid pharmacology. We selected cannabidiol as the proximal cannabinoid for our integrated transcriptomic and metabolomic experiments due to its cost-effectiveness and accessibility in pure form. Figure 1 depicts the decarboxylation mechanism of the acidic cannabinoids Δ9-THCA and CBDA into their neutral forms Δ9-THC and CBD, respectively.

## Results

### CBD treatment incurs a growth inflection after controls have established their stationary phases in yeast cell cultures

**Fig. 2:**
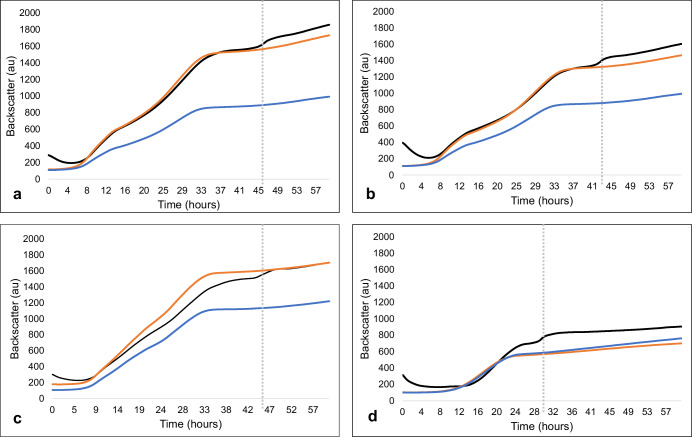
Evaluation of Growth in CBD-administered samples across four experimental setups. In each subplot, the black trace represents the CBD-exposed sample, the orange trace represents the positive control, and the blue trace denotes the untreated sample. Biomass was quantified through backscatter measurements facilitated by an Aquila continual biomass monitoring sensor placed beneath the shake flasks. The dashed gray line in each subplot indicates a critical point of growth inflection for the CBD-treated cultures. **a** Methanol solvent matrix and methanol positive control. Approximately at the 45-h mark, a phase transition can be observed, triggering a second growth phase for the CBD-administered cell cultures. **b** DMSO solvent matrix and DMSO positive control. A metabolic phase transition is again observed. **c** sucrose-fed cultures (methanol solvent matrix). Sucrose was selected because it is an α-linked heterodisaccharide. **d** minimal media (glucose, methanol solvent matrix). In all experiments, there is a persistent growth inflection found uniquely in the CBD-fed cell cultures. All experiments have been replicated a minimum of three times.

We first investigated the effect of cannabidiol treatment on yeast using continual growth monitoring to test if there was any evident growth inhibition or other visible phenotypic effects. To accomplish this, we monitored cell growth of *S. cerevisiae* CENPK2-1C cells inoculated at 0.1 ODU/mL in Erlenmeyer flasks at 30 °C and orbital rotation at 200 rpm continually throughout the logarithmic and latent phases. Treated cells were fed cannabinoids at 0.5 mM at the same time as the cellular inoculation of the flasks. Cannabinoids were introduced in an organic solvent matrix, and appropriate positive controls were implemented (depending on which experiment) to control for the residual effects of the respective organic solvent, along with negative controls with no treatment but identical culture conditions. As a general trend, we observed that the backscatter signal decreases for ~6 h (Fig. 2). However, it is important to note that these growth curves do not represent cell viability density, but signal as measured by a backscatter detector. Given that the highly lipophilic cannabinoids immediately precipitated into globules upon delivery to the aqueous cell media, we hypothesized that this decrease in backscatter represented the assimilation of the cannabidiol into and onto the cells, which would decrease the total number of free particles in the media.

#### A growth inflection point persists in CBD treated yeast cultures independently of the solvent used to introduce the CBD and the nutrient environment

We evaluated the yeast growth curves using a methanol solvent matrix to introduce the cannabidiol into the liquid culture (Fig. 2a). We observed a growth inflection point at around 45 h, which did not occur in either the untreated group or the positive control (0.5% methanol). We thought that this might occur because of a post-diauxic shift to another source of carbon for growth, so we moved to manipulate the nutrient environment of the yeast through a series of experiments to determine if this phenotype persisted across multiple carbon sources.

It is well known that methanol can be metabolized by *S. cerevisiae*, so we decided to further evaluate this effect using DMSO as the solvent matrix (Fig. 2b). In both groups, as the cell growth curves proceeded, it was clear that both the treatment group and positive control grew to a higher density than the negative control, although it was unclear if this was due to carbon utilization of the organic solvent or if the addition of 0.5% organic solvent incurs a physical effect that promotes the growth of the yeast cultures. Given that there is no described mechanism to utilize DMSO as a carbon source in *S. cerevisiae*, we prefer the latter explanation, but further investigation here was outside of the scope of this study. Overall, the total density of the yeast cultures was uninteresting for the study given that the positive controls grew to similar densities. We focused instead on the growth inflection point unique to CBD-fed cultures. After establishing that this growth inflection occurs in both methanol- and DMSO- fed yeast cultures, we concluded that cannabidiol-fed cultures were able to utilize additional carbon and continue growing after the onset of stationary phase in the control groups. Therefore, we investigated this effect further by manipulating the growth media.

We replicated the study in sucrose-containing media (Fig. 2c) to determine if the effect was still visible after switching to a heterodimeric sugar and we observed that the latent stationary phase metabolic shift persisted. We then replicated the study using minimal media yeast nitrogen base (YNB) and once again, observed the same effect (Fig. 2d). These results led us to conclude that the growth inflection point caused by CBD treatment occurs independently of the nutrient environment of the yeast cells.

#### Acidic cannabinoids and cannabidiol analogs with shortened alkyl chains create growth perturbations when treated to yeast cell cultures

Last, we were interested to determine if other cannabinoids behaved similarly to the effects that we observed from cannabidiol treatment. We examined the reproducibility of this effect in yeast cultures which were treated with acidic cannabinoids, as well as the alkyl chain substituted analogs of cannabidiol (CBD-C5), cannabidivarin (CBD-C3), cannabidiorcol (CBD-C1), and cannabidiresoricol(CBD-C0) (Supplementary Information Fig. 1). Interestingly, each acidic cannabinoid incurred a unique effect on the yeast cultures that was distinct from the effect caused by cannabidiol. CBGA-fed cultures behaved like the positive and negative controls except that the beginning of the exponential growth phase was delayed by about 6 h. Each subsequent metabolic shift was similarly delayed, however we did not observe any additional abnormal growth inflections. THCA also behaved almost normally except for a subtle change occurring around 19 h. The pattern of backscatter signal in the CBDA treated cultures was consistent with the controls, however, interestingly, CBDA treatment led to the only example of overall lower backscatter density than the positive and negative controls. Finally, the short-alkyl chain homologs of cannabidiol suspended growth entirely until 40-52 h, at which point growth proceeded normally. Overall, these results demonstrated that each cannabinoid affects yeast culture growth uniquely.

After we determined that cannabinoids exert phenotypic influence on the growth patterns of our yeast, we hypothesized that changes in the central metabolism of the cells were the cause of this observed post-diauxic shift, so we proceeded with a broad, non-targeted integrated omics approach to define the cellular changes that occurred during this growth shift. Therefore, we grew more yeast cultures under continual biomass monitoring and harvested the cultures at the growth inflection point as it occurred. We then harvested RNA from part of the culture suspensions for transcriptomic sequencing and diverted the rest for metabolomic analysis using a UPLC/HRMS.

### Transcriptomics and genomics indicate activation of a pleiotropic drug resistance response upon cannabidiol treatment at the growth inflection point

#### *S. cerevisiae* CENPK2-1C genome sequence enables strain-specific transcriptomic assembly

We first sequenced and assembled the genome of our strain to ensure that our transcriptomic data could be accurately quantified. *S. cerevisiae* CENPK2-1C was chosen for this study because it is a common strain used for metabolic engineering, owing to its deletions of *URA3*, *HIS3*, *LEU2*, and *TRP1*, which can be used as selection markers. However, a well sequenced and annotated genome for this strain is not available. Moreover, as big-data becomes more widely accessible to laboratories, we advocate an approach of using strain-specific curations to validate assumptions that were previously made about yeast genome stability. Given the fundamental importance of having an accurate representation of gene copy number in transcriptomics analyses, we sequenced our internal laboratory strain genome of CENPK2-1C (Euroscarf, *MATa; ura3-52; trp1-289; leu2-3,112; his3-Δ1; MAL2-8*^*c*^*; SUC2*) using Nanopore long reads for assembly and Illumina short reads for polishing. After filtering reads less than 1000 bp, our Nanopore run sequenced 477,258 total reads. The mean read length was 10,566.8. Additional information, including a histogram of the read lengths and the Nanoplot summary statistics, is available in the supplementary material. After Flye assembly^29^, our genome consisted of 28 contigs with an N50 of 800.5 Kbp and a genome size of 11.98 Mbp. Upon realigning the reads to the assembly using Minimap2^30^, we observed an average coverage depth of 958. As these quality checks were satisfactory, we moved to polish the assembly with ONT’s medaka© (using long reads) and then with pilon^31^ (using trimmed short Illumina reads). Upon assembly and polishing, a BUSCO^32^ analysis indicated 99.4% completeness of the assembly (Supplementary Information Fig. 6). Last, we used Funannotate (software, open access) and the annotation transfer tool in Geneious to annotate a total of 4459 genes. To evaluate and benchmark the differences between the genomes, we created a 2D dot plot using Nucmer and DNAdiff, the results from which we have included in the supplementary material as Supplementary Fig. 62 and Supplementary Table 6. We identified a total of 23468 SNP’s, 6524 indel’s, 12 relocations, 23 translocations, and 305 insertions. While these findings indicate an overall high degree of similarity evidenced by the genome alignments, the differences are nevertheless relevant to our transcriptomics study.

#### Differential gene expression analysis shows eight genes are overexpressed in response to cannabidiol

We pursued differential gene expression analysis of our CBD-fed and MeOH-fed positive controls because we wanted to determine which genes were being expressed at the time point of the growth inflection. After sequencing, we evaluated our transcriptomic data sets using Nanoplot to ensure that read counts would be sufficient for quantitative analysis. The Nanoplot data for the transcriptomic cDNA libraries is provided in the supplementary information. A total of 1,448,164 reads, derived from both CBD-fed and positive control (MeOH) group libraries, were stranded using the UNAGI^33^ pipeline and assembled to the *S. cerevisiae* CENPK2-1C genome using Minimap2. In essence, UNAGI is a strand-aware transcriptome assembly pipeline designed for handling cDNA reads generated by Nanopore sequencing. Subsequently, gene expression quantification was performed using Geneious. The alignments resulted in read count tables, encompassing the analysis of 4435 genes. A significant subset of these genes was associated with biosynthetic processes, prompting the inclusion of an additional set of reads from a mid-logarithmic growth phase cDNA library data set. This additional set comprised 2,966,571 reads also assembled to the S. cerevisiae CENPK2-1C genome, providing a more precise perspective for our DESeq2 analysis.

**Fig. 3:**
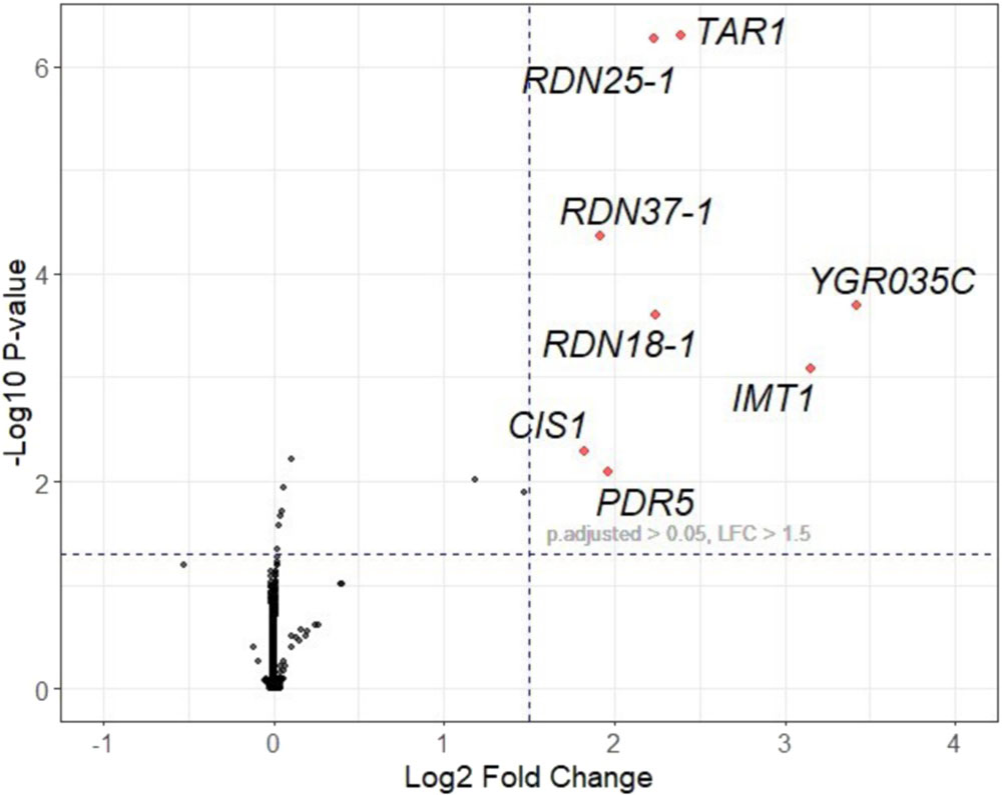
Volcano plot visualization of differential gene expression analysis. Points which are labeled and shown in red represent genes that were identified to have greater than 1.5 log_*2*_ fold change (LFC) and an adjusted p-value less than 0.05. Most transcripts were expressed at or near the same level. Eight transcripts exceeded the significance threshold; *CIS1, RDN37-1, PDR5, RDN25-1, RDN18-1, TAR1, IMT1, YGR035C*.

Our comparative analysis of read sets was performed using DESeq2^34^ (*n* = 6), where the CBD-fed group was the experimental focus and was compared against the MeOH-treated group sampled in stationary phase at the same time as the experimental group (positive control) and an untreated, midlogarithmic-phase group (negative control). DESeq2 was selected for its ability to effectively handle small sample sizes using a shrinkage estimator for dispersion and fold changes. We hypothesized that the mean fold change should be 0 because most genes should be expressed at the same levels. We observed a mean fold change of 0.006, which confirmed that DESeq2 was an appropriate method to compare our data sets given our experimental design. A cut-off for significance was established at a log2foldChange of >1.5 and an adjusted p-value of <0.05. Significant overexpression was observed for the following genes: *CIS1, RDN37-1, PDR5, RDN25-1, RDN18-1, TAR1, IMT1, YGR035C*. These results are visually represented in a volcano plot (Fig. 3).

#### Pleiotropic drug resistance is expressed in CBD-treated yeast

This set includes three notable genes which are a part of the pleiotropic drug resistance^35^ response in yeast; *PDR5*^36^, *CIS1*^37^, and its paralog *YGR035C*. Little is known about *CIS1*, other than it is under regulatory control of the PDR-involved transcription factor Yrr1p and it is localized to mitochondria. It shares 21.6% sequence identity and little structural similarity with its paralog *YGR035C*^38^ (Supplementary Information Fig. 8). *YGR035C* is also under the regulatory control of PDR-linked transcription factors Yrm1p and Yrr1p. Interestingly, WoLFPSORT predicts subcellular localization to the nucleus (Supplementary Information Table 4). While the two proteins have little in common, there are 26 conserved residues across the peptides and a 15 amino-acid region which contains 12 conserved residues, *PXKITRYDLXKAAXE*. The alignment and the predicted structures with the residues highlighted in red are available in the supplementary information.

In contrast to *CIS1* and *YGR035C*, much is known about the promiscuous ABC steroid and cation transporter *PDR5*^39^*. PDR5* is localized to the plasma membrane and is under the positive regulation of the transcription factors Pdr1p and Pdr3p^40^, however many transcription factors have been shown to induce a *PDR5* response both positively and negatively. *PDR5* is known to facilitate removal of drugs, steroids, antifungals and phospholipids from yeast cells and has been implicated in many studies as a critical part of the pleiotropic drug response^41,42^. *PDR5* is known to facilitate cation transport^43^ and lipid translocation^44,45^. *PDR5* is an important gene which has been used as a model to study the accumulation of drugs into yeast cells and the induction of pleiotropic drug resistance^46,47^. *PDR5* has several homologs, including *PDR15* and *PDR10*. In our dataset, no other genes involved in PDR or vacuolar sequestration were overexpressed. These results suggest that under normal growth conditions, *PDR5* is targeted and specific towards cannabidiol.

#### Mitochondrial regulation and cellular biosynthetic processes are expressed at the growth inflection point

The remaining significant genes in the dataset were *TAR1, RDN25-1, RDN37-1, RDN18-1*, and *IMT1. TAR1* and *RDN25-1* are opposite adjacent neighbors in the genome, and their expression is tightly coregulated^48^. *TAR1* is a mitochondrial activity regulator^49^, and the remaining genes are rRNA’s and the tRNA *IMT1*. These genes, which are related to biosynthetic processes, remain significant after we controlled for the influence of cellular proliferation using an RNA library collected during normal mid-logarithmic growth phase to compare to our treatment group in our DESeq2 analysis. One possible explanation is that they may be connected to a change in the source of carbon for the cell cultures, consistent with a post-diauxic shift.

### Metabolomics corroborates a post-diauxic shift model and identifies unique and overabundant lipids at the growth inflection point

#### Cannabidiol treatment instigates an intracellular metabolic response in *S. cerevisiae* CENPK2-1C at the observed phenotypic change in a growth curve

**Fig. 4:**
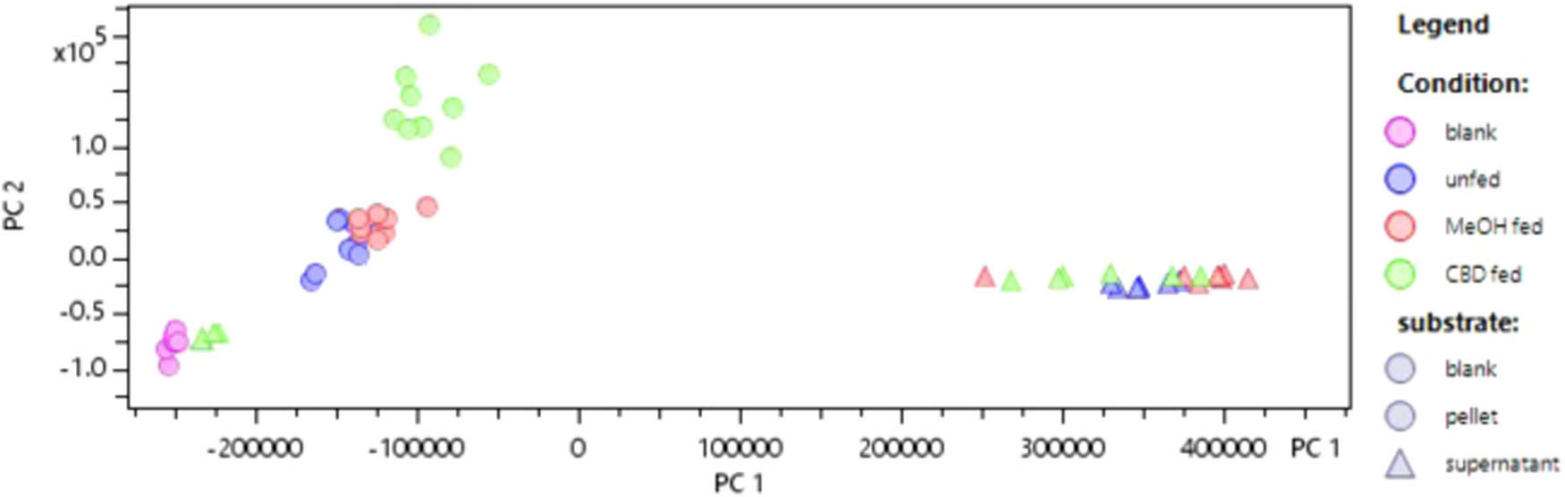
Visualization of LC-MS(qTOF) untargeted metabolomics data with PCA. PC1 is plotted against PC2. 30 principal components explained a variance (*R*^2^) of 0.957.

We were interested in metabolomics because we hypothesized that changes in central and secondary metabolism would underpin the cause of the observed post-diauxic shift. Our questions regarding metabolism were if samples within treatment groups were similar to each other, especially if CBD-treated samples shared high similarity; and which compounds were overabundant within CBD-fed samples. Principal Component Analysis (PCA) revealed distinctive shifts in the intracellular metabolite composition of *Saccharomyces cerevisiae* upon cannabidiol (CBD) treatment. The cell pellets demonstrated a higher degree of clustering influenced by CBD treatment compared to supernatants, suggesting a pronounced intracellular response to CBD feeding. We base this conclusion on the clustering patterns we observed in our PCA, where supernatants clustered tightly regardless of their condition while cell pellets demonstrated separation across conditions (Fig. 4). This PCA showed that 30 principal components explained a variance (*R*^2^) of 0.957 indicating a robust data model.

After PCA, we conducted a two-way Analysis of Variance (ANOVA), which facilitated the discovery and annotation of numerous significant metabolites. Out of the 2798 spectra identified in the entire data set, 417 were found in the solvent blanks, leaving 2381 spectra that we found in our supernatant and cell extracts. We wanted to know which of these were abundant in CBD-fed cell pellets, so we removed the supernatant samples from the dataset and ran our ANOVA. 480 spectra showed significant alpha values in a two-way ANOVA, with condition (CBD-fed, MeOH-fed, unfed, or blank) as the experimental group and substrate (pellet or blank) as the fixed factor. Each condition was tested in biological triplicate (n = 9) with each biological replicate sampled three times for technical replication. We used ANOVA to evaluate our data as it enabled simultaneous comparison across multiple conditions and accounted for variable subsets within each condition and allowed us to identify individual compounds which were important to the intercellular response from cannabidiol. Among the significant compounds, we were especially interested in 48 spectra because of their overabundance in the CBD-fed cell pellets. We determined overabundance based on a comparison of their intensity counts to the other samples. These intensity plots are included in the supplementary information. From these 48 spectra, twenty non-cannabidiol compounds were identified via HRMS parent ions and in-source fragments, all of which were within 0.003 of the theoretical m/z value. Detailed information regarding the retention times, m/z, parent masses (M), and molecular formulas are presented in Table 1. A compound of particular note was 1-docosanoyl-glycero-3-phosphoserine, which was present exclusively in CBD-fed cells (Fig. 6). The identification of 1-docosanoyl-glycero-3-phosphoserine was substantiated by in-source fragments. These metabolites were then used for a KEGG pathway analysis.

**Table 1:** Compounds marked with ^a^ are considered central metabolites and compounds marked with ^b^ act as central or secondary metabolites, depending on the pathway in which they participate

Significant Metabolites found in *Saccharomyces cerevisiae* cell pellets after CBD treatment				
RT (min)	m/z (-)	M.	Name	Formula
1.8	283.2624	284.2696	Stearic acid^a^	C_18_H_36_O_2_
1.82	281.2467	300.2644	2-Hydroxystearic acid	C_18_H_36_O_3_
1.84	297.2441	298.2514	9-Hydroxy-12-octadecenoic acid	C_18_H_34_O_3_
1.98	580.3603	581.3676	1-docosanoyl-glycero-3-phosphoserine	C_27_H_51_N_9_OS_2_
2.06	186.1131	187.1204	8-Amino-7-oxononanoate	C_9_H_17_NO_3_
2.5	146.0279	147.0352	Thiomorpholine 3-carboxylate	C_5_H_9_NO_2_S
3.14	164.0718	165.0791	L-Phenylalanine^a^	C_9_H_11_NO_2_
3.15	119.0353	120.0426	D-Erythrose^a^	C_4_H_8_O_4_
3.36	130.0879	131.0952	L-Leucine^a^	C_6_H_13_NO_2_
3.42	203.0813	204.0886	L-Tryptophan^a^	C_11_H_12_N_2_O_2_
3.58	130.0879	131.0952	L-Isoleucine^a^	C_6_H_13_NO_2_
3.76	148.0444	149.0517	L-Methionine^a^	C_5_H_11_NO_2_S
4.18	245.0429	246.0502	Glycerophosphoglycerol^b^	C_6_H_14_O_8_P
4.25	180.0658	181.073	L-Tyrosine	C_9_H_11_NO_3_
4.55	241.0848	242.0921	Thymidine^b^	C_10_H_14_N_2_O_5_
4.63	179.0556	180.0629	D-glucose^a^	C_6_H_12_O_6_
4.65	344.0393	345.0466	Nucleotide Monophosphate^a^	C_10_H_12_N_5_O_7_P
6.2	88.03968	89.04696	L-Alanine^a^	C_3_H_7_NO_2_
7.59	154.0627	155.07	L-Histidine^a^	C_6_H_9_N_3_O_2_
9	214.0475	215.0548	*sn*-Glycero-3-phosphoethanolamine^a^	C_5_H_14_NO_6_P

#### Central and secondary metabolites reveal enrichment of pathways related to cell growth

**Fig. 5:**
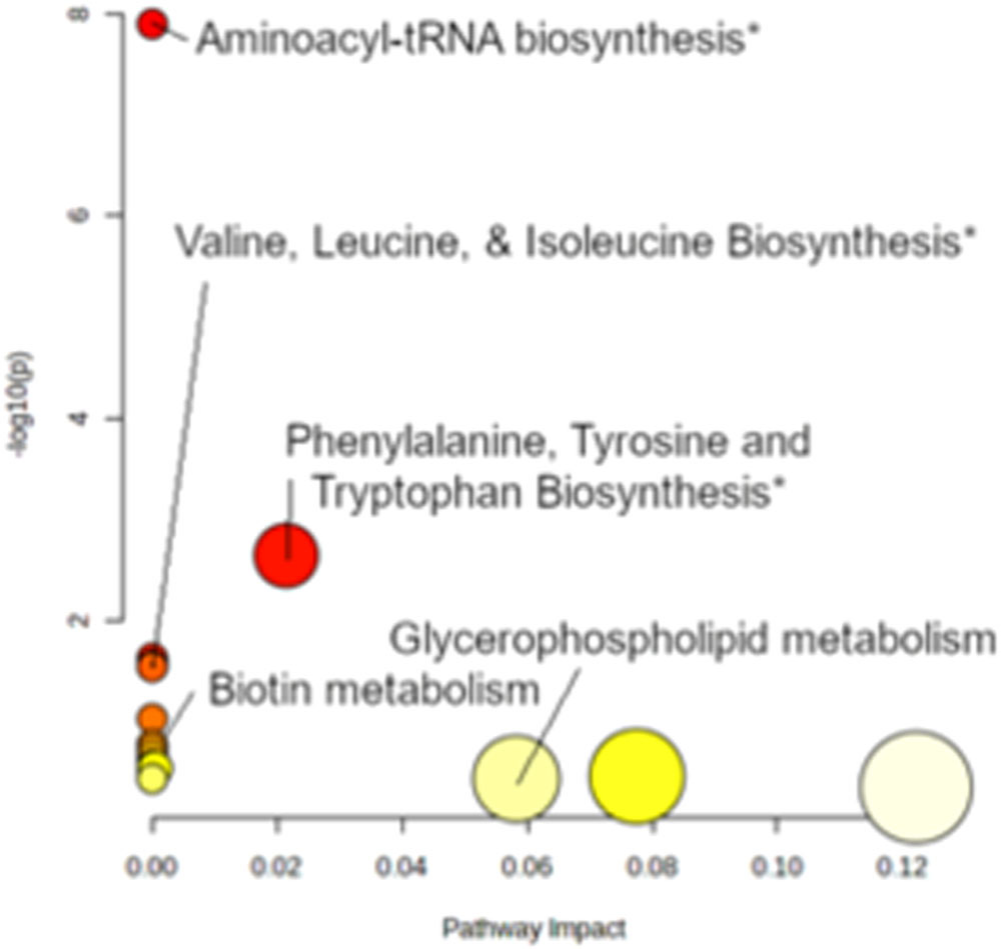
Comprehensive Analysis of LC-MS(qTOF) metabolomics data reveals key pathways. Asterisks denote pathways of significant alteration. Predominant among these is aminoacyl-tRNA biosynthesis, highlighted by an elevated presence of metabolites including phenylalanine, leucine, tryptophan, methionine, tyrosine, alanine, and histidine. This observation aligns with transcriptomic data (Fig. 3), particularly the upregulation of *IMT1*. The biosynthesis of branched-chain amino acids (valine, leucine, isoleucine) is also indicated by the notable levels of leucine and isoleucine. Phenylalanine, tyrosine, and tryptophan biosynthesis are further corroborated by their significant presence. While biotin metabolism and glycerophospholipid metabolism did not show significant changes, the detection of 8-Amino-7-oxononanoate and specific lipids (unrepresented in the KEGG database) such as 9-Hydroxy-12-octadecenoic acid and 1-docosanoyl-glycero-3-phosphoserine suggests underlying activity. Detailed data are presented in Supplementary Table 5.

The significant metabolic pathways delineated by our KEGG pathway analysis (Fig. 5) yielded vital insights into the cellular machinations instigated by CBD exposure. We observed upregulation in pathways such as aminoacyl-tRNA biosynthesis; phenylalanine, tyrosine, and tryptophan biosynthesis; valine, leucine, and isoleucine degradation and biosynthesis, as well as ubiquinone and other terpenoid-quinone biosynthesis. The convergence of these pathways, particularly those involved in amino acid metabolism and the biosynthesis of essential coenzymes, suggests a response of primary metabolism to CBD treatment, with potential implications for cellular growth and stress adaptation mechanisms. Overall, an enrichment of tRNA biosynthesis corresponds tightly to transcriptomics dataset, which found significant overexpression of the tRNA initiation factor *IMT1*. In parallel to the changes in amino acid metabolism and glycerophospholipid metabolism, we can infer that central carbon metabolism does in fact showcase that a cellular growth response takes place at the sampled time point, corroborating the post-diauxic shift hypothesis.

#### Cannabidiol treated *S. cerevisiae* CENPK2-1C uniquely produce a 22 carbon monoacylglycerophosphoserine

**Fig. 6:**
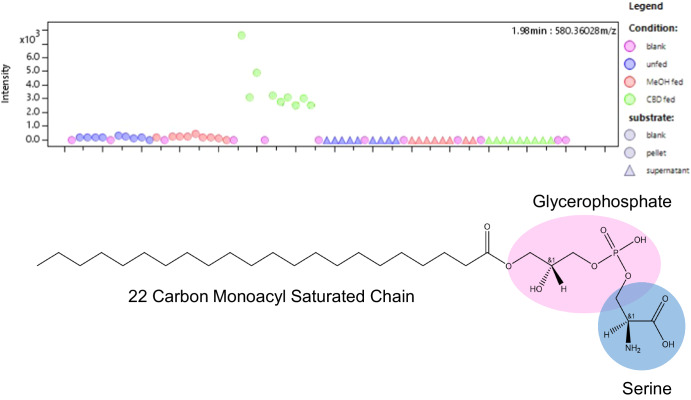
Intensity (y-axis, a.u.) plot of the m/z peak at 580.36028 across all samples (x-axis) in the analysis. (Top) This compound was found only in cell pellets from CBD-treated samples, indicating that cannabidiol initiates the production of this compound in vivo. (Bottom) A visual description of 1-docosanoyl-glycero-3-phosphoserine, the identity of the compound associated with the M. 581.3676 (see Table 1). 1-docosanoyl-glycero-3-phosphoserine is a monoacylglycerophophatidylserine with 22 carbons in the alkyl chain. We predict it is associated with the plasma membrane because it is a glycerophospholipid, a group of lipids commonly associated with plasma membrane construction.

We report the discovery of high production of 1-docosanoyl-glycero-3-phosphoserine which was exclusive to our CBD-fed cells (Fig. 6). We confirmed the identity of this molecule with four fragment matches we found in Metfrag. Moreover, we report the existence of an additional fragment at 528.28483 m/z (negative mode), which is made obvious by a shared abundance pattern across all samples for this spectrum across all significant spectra we identified as this retention time (see Supplementary Information Fig. 2). Further, when we ran an independent analysis using reverse-phase chromatography to check for cannabidiol metabolites, we replicated the discovery of this compound (however, metabolites of cannabidiol were not identified- see Supplementary Information Fig. 3). Within the cell-pellet analysis, glycerophosphoglycerol and *sn*-glycero-3-phosphoethanolamine were exciting to identify because it furthers the body of evidence which points towards dynamic lipidomic remodeling in the CBD-treated cells and might be related to the production of 1-docosanoyl-glycero-3-phosphoserine. While the pathway analysis did not reach a significant p-value for the category of glycerophospholipid metabolism, we note that this analysis excluded 9-Hydroxy-12-octadecenoic acid and 1-docosanoyl-glycero-3-phosphoserine from the input, as they were not available in the KEGG database. Accordingly, we conclude that this result is currently inconclusive and warrants further investigation.

### Knock-out and complementation of PDR5 results in deletion-and-rescue of the post-diauxic shift Phenotype

To further underpin the contribution of the overexpressed transcripts to the observed phenotypic changes in our study, we performed a knock-out and complementation assay on *PDR5* and a knockout assay of *CIS1*. *CIS1* was deleted and selected for using a uracil marker. *PDR5* was knocked out using a kanamycin marker, or for complementation was knocked out and selected for using a *PDR5*-kanamycin insertion cassette.

In continual biomass monitoring experiments, Δ*cis1*::*URA3* behaved as the wildtype, demonstrating that the post-diauxic shift phenotype occurs independently of the presence of a functional copy of *CIS1* in the genome. However, Δ*pdr5*::*KAN* lost the associated post-diauxic shift and Δ*pdr5*::PDR5-*KAN* regained the function. Data are shown in Supplementary Information Fig. 63.

## Discussion

In this paper we characterize the impact on yeast cell cultures by CBD with the goal of improved understanding of cannabinoid biosynthetic YCF systems. We observed that CBD instigates a cellular response (Fig. 2), which is believed to be the result of a post-diauxic shift to a carbon source that is uniquely available in the CBD-fed cultures.

DESeq2 analysis showed a mean near 0 and thus demonstrated that despite the differences in time at which this phenotypic response mounts, the transcriptomic conditions were well-controlled during our study. While there was some variability in the time at which this response occurred, we have successfully compared the phenotype across several conditions by sampling at the point of concavity of a growth-inflection in a growing culture of cells.

The principal artifacts of the yeast response to CBD include lipidomic and transcriptomic changes, where a 22 carbon monoacylglycerophosphoserine is uniquely identified in CBD-treated cell pellets and stearic acid and a hydroxylated stearate are overrepresented. We also identified the lipid-biosynthesis precursor molecules, glycerophosphoglycerol and *sn*-Glycero-3-phosphoethanolamine abundantly in the CBD-fed pellets. In this dataset, the parent fragment of 581.36756 was used to identify 1-docosanoyl-glycero-3-phosphoserine using LipidMaps.org. Subsequently, the shared-retention time (2 min) m/z peaks at 552.32934, 538.31315, 478.29205, and 450.26152 all corresponded to fragment matches in MetFrag. We argue that the peak at 528.28483 is also a fragment of this compound based on the shared abundance of intensity in comparison with other fragments associated with this compound at the 2-min retention time (see Supplementary Information Fig. 2). 1-docosanoyl-glycero-3-phosphoserine has not been reported in yeast before, but given the strong support for its identity with fragment matching we are confident in reporting this lipid in our samples. There is no support for this molecule as an MS artifact which occurs during the ionization process, which we believe to be unlikely.

It is possible that 1-docosanoyl-glycero-3-phosphoserine in CBD-fed cells accumulates to regulate the properties of the plasma membrane. It has been previously reported that yeast membranes are primarily composed of 16-C and 18-C diglycercophospholipids. If 1-docosanoyl-glycero-3-phosphoserine is integrated into the plasma membrane, then its singular alkyl chains would change the properties of the fluid membranes. This discovery may support the cellular stress concept since lipids preserve membrane integrity and fluidity. The significance of the serine backbone is not yet understood or well-researched within the yeast community, as phosphatidylserines in yeast are mostly understood as precursors of phosphatidylethanolamines. While not answerable by our data, it is possible that 1-docosanoyl-glycero-3-phosphoserine might be a constituent of lipid rafts, which are specialized subdomains of the plasma membrane and are known to be associated with pleiotropic drug resistance enzymes embedded within the plasma membrane. This is an interesting question which could be addressed with further research.

The pathway of 1-docosanoyl-glycero-3-phosphoserine generation remains unclear. The accumulation of 1-docosanoyl-glycero-3-phosphoserine in the yeast cell is likely a polygenic trait and requires many metabolic reactions from production of a 22-carbon long chain fatty acid to the attachment of the glycerol and then a phosphoserine backbone, but to date no biochemical pathways which exist in yeast explain its synthesis. While establishing the genetic basis for this molecule is outside of the scope of this study, we will briefly explore two potential hypotheses. First, it is built up anabolically as a monoacylglyceride^50–56^. This hypothesis is uncertain because affinity for a monoacylglycerol at each step in normal lipid biosynthesis is unlikely. Moreover, in this data, none of the genes that would be useful for lipid biosynthesis were perturbed either positively or negatively at the transcriptional level. Alternatively, the molecule could be a catabolic byproduct of a fatty-acid oxidation process used to harness energy during the hypothesized post-diauxic shift. Numerous central metabolites and tRNA’s corroborate this hypothesis. Further work, presently outside of the scope of the study, utilizing lipidomics could help to identify a parent molecule of 1-docosanoyl-glycero-3-phosphoserine to confirm this hypothesis.

In yeast, phosphatidylserines are a necessary intermediate to produce phosphatidylethanolamines, which are an abundant phospholipid constituent of the plasma membrane, via phosphatidylserine decarboxylases^57^. We identified an enrichment of glycerol-3-phosphoethanolamine, however we did not identify a significant association of any long-acyl-chain phosphoethanolamines to our CBD treated samples, which suggests that the metabolic route to phosphoethanolamines does not proceed from 1-docosanoyl-glycero-3-phosphoserine. Alternatively, the catabolic route from a tri- or di- acylphosphatidylserine to a monoacylphostphatidylserine would only require a lipase. While not identifiable at this time, the yeast genome contains several uncharacterized putatative lipases, such as LIH1, which might be involved in this process. Biochemical elucidation of the metabolic process that produces 1-docosanoyl-glycero-3-phosphoserine would enable a precise knock out of this molecule, which would redirect carbon and cellular energetics towards molecules of interest. The degradation route stands out as a compelling alternative hypothesis because it could be the cellular energy source that enables a diauxic growth pattern as we have seen in our data.

While it is an interesting research question to elucidate the molecular basis of 1-docosanoyl-glycero-3-phosphoserine, we emphasize that a systems approach should be considered when determining how to perturb unnecessary very-long-fatty-acid chain accumulation in a cannabinoid-biosynthetic yeast strain, which is heavily influenced by the strain engineering itself because overexpression of one gene can influence the cell systemically. This renders a limitation to our study, that we have not implemented the underlying genetic architecture necessary for a cannabinoid YCF. This is, however, by design, because genetic manipulation would confound the response that would be seen in a normal yeast cell against exposure to cannabinoids. We therefore reiterate that further studies towards these results should be done in yeast systems wherein heterologous pathways are already inserted and that these results should be reexamined at each iterative step towards cannabinoid optimization. While direct disruption of any one gene which is involved in the biogenesis of 1-docosanoyl-glycero-3-phosphoserine could be useful for determining how this lipid is generated by yeast cells, it also needs to be contextualized as part of a cannabinoid biosynthetic system where constraints on total protein content and energetic capacity should also be considered. Most importantly, bioengineers should be aware that cannabinoid accumulation causes a pleiotropic drug resistance response and assay for 1-docosanoyl-glycero-3-phosphoserine and other very long chain lipids in their unique yeast species and strains.

Transcriptomically, we identified the overexpression of four compelling significant transcripts; *PDR5, YGR035C, CIS1*, and *TAR1*. *YGR035C* and *CIS1* are reported paralogs but have only 21.6% sequence identity at the amino acid level (Supplementary Information Fig. 8). Despite this, they do share many conserved residues and appear to have a conserved region of 15 amino acids that contains only three substitutions, which calls into question if this is a functional region and if these two proteins still share functional similarities. If so, they likely have diverged to act within different subcellular localities, as *CIS1* is localized to the mitochondria and *YGR035C* is predicted to be localized within the nucleus (Supplementary Information Table 4). It is not within the scope of this paper to assign a functional purpose to these proteins, although this is another interesting research gap.

*PDR5*, a well characterized protein involved in pleiotropic drug response, is known for its promiscuity to xenobiotics and responds to exogenous drugs, steroids, antifungals, and phospholipids. We have also demonstrated, through knockout and rescue, that *PDR5* is a determinant gene in the presence of the post-diauxic shift phenotype. As shown in Supplementary Fig. 63, reinsertion of PDR5 into a *Δpdr5* under a pTEF1 promotor not only recovered the post-diauxic shift phenotype, but enabled higher biomass accrual over the duration of the experiment. As pTEF1 is a very strong promoter it appears that higher PDR5 expression correlated to increased growth, however the underlying mechanism for this is not defined in this study. In this study, we hypothesize that the shared structural motifs between cannabidiol and steroids, such as six-carbon ring groups, explain why *PDR5* was a part of this cellular adaptation. Notably*, PDR10, PDR15*, and *SNQ2* were not overexpressed, which suggested that Pdr5p was the only enzyme in this family with binding affinity for cannabidiol. Important to the study, we note that *PDR5* is an ABC transporter which consumes energy, diverting potential activity from cannabinoid biosynthesis and translocates lipids, which perturbs cellular lipid metabolism. We recognize that export of cannabinoids by Pdr5p may be useful for the bioprocessing strategy for some engineers because an export and capture technique could potentially be applied in bioprocessing. However, we advise that *PDR5* activity be incorporated into the modeling to ensure that fluxes and total protein weight are correctly calculated, particularly in the context that PDR5 is involved in the metabolism of the post-diauxic shift in the presence of cannabinoids.

Regarding *TAR1* and the remaining upregulated transcripts, upregulation of mitochondrial activity and ribosomal RNA genes might suggest increased metabolic demands because of a shift towards a new carbon source. The pathway analysis shown in Fig. 6 corresponds to the upregulation of these genes because we see that the cell cultures are overexpressing central metabolites that are necessary for tRNA biosynthesis, amino acid metabolism, and the glycerophospholipid metabolism. One interesting possibility is that if 1-docosanoyl-glycero-3-phosphoserine is, in fact, produced via lipid degradation by a lipase, then its’ parent molecule might be the energy source which enables this cellular growth response.

We have deliberately kept the scope of our paper fixed within the impact of CBD on yeast, however, it is important to note that the structural diversity of cannabinoids as a group prevents cannabidiol from serving as a comprehensive representative of cannabinoids. While a limited conclusion, it is still plausible that similar mechanisms would be employed to overcome growth deficits resulting from cannabinoid accumulation, however, these must be determined empirically throughout the development process for each unique cannabinoid-biosynthetic YCF. We emphasize to the reader that these conclusions are a starting point for bioengineers who wish to improve the accuracy of their metabolic models or improve intracellular carbon flux towards cannabinoid production. The findings of this paper should be used as a guideline for bioengineers to apply to their own particular use-cases.

In the field of cannabinoid bioengineering, the implications of our findings are substantial. We have shown that a pleiotropic drug resistance response mounts in yeast which are exposed to CBD at a reasonable concentration of 0.5 mM. We based this concentration off existing metabolic models. This would sponge up valuable bioengineering substrates such as ATP, electron carriers, CoA’s, and fatty-acid metabolism precursors. To mitigate this risk, creators of cannabinoid-biosynthetic yeast or cannabinoid-biotransforming yeast (such as researchers who seek to glycosylate cannabidiol via cellular transformation in large scale bioreactors), could assay to determine the quantity of these overexpressed transcripts and then manipulate their quantity through a transcription factor knock out strategy. Researchers should expand the scope of the ABC transporters that they screen for, as other PDR transporters might be expressed depending on the conditions of the culture, such as low pH, high salinity, or changes to temperature. These include *PDR15* and *PDR10*. They should evaluate the expression of these genes for each new cannabinoid they introduce into their systems. Additionally, stearic acid and/or LPS(22:0) (or glycerophosphoglycerol, *sn*-Glycero-3-phosphoethanolamine, and other precursors, if more cost effective) could be added to the medium as a media supplement in order to potentially influence the response of the yeast to the CBD treatment. For the purposes of biotechnology, it will be necessary to conduct additional research to fully comprehend and exploit the potential modifications. Bioengineers should consider the trade-off between cell growth and energy usage by the cell in the pleiotropic drug response. It is not currently understood why cannabinoid-biosynthetic yeast continues to hold titers which are substantially lower than the theoretical yield of 300 mg/L, however the plausible explanations have been reviewed at length. In short, the efficiency of candidate prenyltransferases is the most common suggestion for how to improve cannabinoid yield in yeast cultures. However, despite previous publications reporting the induction of pleiotropic drug resistance and lipid membrane remodeling in response to heterologous yeast xenobiotics, these traits have not yet been proposed as potential obstacles to cannabinoid YCF’s. We believe that the results from this paper present a compelling argument towards the necessary consideration of energy and carbon flux in cannabinoid-biosynthetic yeast. To contextualize this conclusion in the field of systems bioengineering, we have showcased another example where integrated omics provides necessary insights for accurate systemic modeling. This is a growing sense of understanding in the field, which was coined the “design- build- test- learn” model by Chen. et al. ^58^ The relationship in systems biology between theoretical modeling and empirical validation is steadily growing, which is enabled by the increasing accessibility of -omics technologies and the rapidly improving algorithms for analyzing these large sets of data. In this sense, we hope that the conclusions of this paper will extend beyond the cannabinoid-YCF community and be considered by YCF engineers across the field.

## Materials and methods

### Kits and reagents

All reagents were purchased from Sigma unless stated otherwise. Organic solvents were gradient grade and purchased from VWR, except when used for analytical purposes, in which case they were Honeywell ChromaSolve® MS grade organic solvents. All Oxford Nanopore Technology kits and flow cells were purchased directly from Oxford Nanopore. All NEB reagents were purchased directly from NEB. Cannabinoids were purchased as analytical standard grade. Short-alkyl chain homologs of CBD were synthesized and purified according to the procedure defined in our previous publication^59^.

### Experimental design

We monitored cell growth of *S. cerevisiae* CENPK2-1C cells inoculated at 0.1 ODU/mL in Erlenmeyer flasks at 30°C and orbital rotation at 200 rpm continuously throughout logarithmic and latent phase. Treated cells were fed cannabinoids at 0.5 mM at the same time as cellular inoculation of the flasks. Cannabinoids were introduced in an organic solvent matrix, and appropriate positive controls were implemented to control for the residual effects of the organic solvent such as carbon utilization of methanol.

All cultures were grown in 250 mL Erlenmeyer flasks. All cells were cultured in YPD with 2% glucose unless stated otherwise. Cultures grown in minimal media were grown in YNB, with all amino acids, and 2% glucose. The *S. cerevisiae* CENPK2-1C yeast strain was inoculated into the sterile media at a starting OD600 of 0.1. All cannabinoids were added from a 100 mM preparation in methanol (or dimethyl sulfoxide, DMSO). Cannabinoid was added to the media at a final concentration of 0.5 mM at the beginning of the culture, and the culture was mixed well and distributed into sterile culture tubes or flasks. The cultures were incubated at 30 °C with agitation for 60+ h. Light backscatter in the flask was monitored continually by the Aquila biomass monitoring system. The growth rates of the cultures were determined by analyzing the data obtained from the Aquila system. Experimental points of interest were determined based on visual inspection of the backscatter curve and growth-shift events as determined by sudden increases to the rate of change of the backscatter curve.

### Genome sequencing and assembly

For growth, harvest and spheroplastization, the yeast cells were grown in liquid culture to mid-logarithmic stage. They were then centrifuged and resuspended in sterile water and softening medium (100 mM Hepes-KOH at pH 9.4 and 10 mM DTT). After incubating for 15 min, the cells were centrifuged and resuspended in spheroplasting medium (YNB with amino acids, 2% glucose, 50 mM Hepes-KOH at pH 7.2, 1 M sorbitol), to which zymolyase was added. The cells were then incubated for 60 min at 30 °C and centrifuged again. After spheroplastization, the cells were lysed in a buffer containing NaCl, followed by NEB lysis buffer and with Proteinase K. After 1 h of agitation, lysates were treated with RNase A (NEB) and incubated overnight. Cells were then centrifuged to pellet cell debris, and treated with NEB Protein Separation solution, and centrifuged at 16000 *g* for 10 min. Samples were then transferred into ice cold ethanol for DNA precipitation. Strands were collected with a hooked glass rod and transferred into a new tube for solubilization in pure water for 24 h. Where applicable, DNA was further cleaned up using a Zymo DNA Clean and Concentrator kit per manufacturer’s directions. Genomic libraries were prepared using Oxford Nanopore Technology using ONT-LSK-SQK109 and were prepared for Illumina sequencing Illumina TruSeq PCR-Free DNA Sequencing kit. Sequencing was then carried out using an Oxford Nanopore Technology MinION MK1C, with an R9.4 flow cell, and an Illumina MiSeq 600c, respectively. Basecalling for ONT was performed using ONT Guppy© with the super-accurate basecalling algorithm.

Super-accurate basecalled FastQ files and Illumina data files examined for quality using FastQC and Illumina files were trimmed with Trimmomatic^60^. Cropping was done until per base sequencing qualities were at least 20 and per base sequences scores indicated approximate equality of A to T and G to C throughout the reads. The resulting Nanopore reads were then analyzed using Nanoplot (Supplementary Information). Nanopore reads less than 1000 bp were filtered. Oxford Nanopore reads were then assembled using Flye under default parameters.

All contigs from the Flye assembly were annotated first with Funannotate, using default parameters, where all identified features were named under an arbitrary naming schema, and were also annotated with transferred annotations based on 100% identity using Geneious©. The genome annotation was then validated and corrected with the help of the gff3 online validation tool from http://genometools.org. To compare the genome S. cereivisae CENPK2-1C with the S. cerevisiae S288C R64 reference genome, we aligned the genomes using Nucmer^61^ (under default parameters except for a minimum cluster length setting of 100 and a maximum gap distance of 500) and created a MUMmer 2D dot plot plot with filtering and fattest alignment options set to yes. We also used the DNAdiff tool to quantify the differences between the genomes.

### Transcriptome sequencing and assembly, analysis parameters

Cannabinoid treated yeast cultures were grown until the backscatter monitoring system indicated that an inflection point in the growth curve was occurring, at which point we stopped the cultivation and snap-froze cell pellets from 15 mL of liquid culture with liquid nitrogen. A positive control group of methanol-fed yeast cells were simultaneously cultured and harvested at the same time as the control group. Each treatment was cultured in three biological replicates. Cells were stored at −80 °C until RNA harvest. RNA was extracted using the Trizol/chloroform method, where cell pellets were thawed in one mL of Trizol reagent and transferred into 2 mL Eppendorf tubes containing glass beads. Cells were then lysed mechanically via vortexation for 5 min in a 4 °C room. RNA was recovered in two treatments of chloroform and precipitated with isopropyl alcohol, then washed with 70% ethanol. cDNA libraries were prepared and barcoded according to the ONT LSK-PCB109 protocol and sequenced using an ONT MinION MK1C using an R9.4 flow cell. Each biological replicate from both treatment groups were barcoded individually and sequenced simultaneously on the same flow cell.

All reads were basecalled using super accurate basecalling in ONT Guppy©. Passed reads were then examined using Nanoplot© for sufficient read quantity and quality before assembling using UNAGI. Stranded reads produced from UNAGI were then backmapped to the *S. cerevisiae* CENPK2-1C genome using Minimap2 and read counts were evaluated under default parameters in Geneious. Read count files were exported and analyzed in R using DESeq2. The reads from CBD-fed and positive control were compared. However, to rule out the possibility that the significant transcripts were only identified due to specific cellular growth conditions, we included an additional dataset, which was collected during the mid-logarithmic growth phase. Figures were produced in R.

### Metabolomics

Cannabinoid treated yeast cultures were grown until the backscatter monitoring system indicated that an inflection point in the growth curve was occurring, at which point we stopped the cultivation and extracted metabolites. One mL of yeast cell culture was transferred to a microcentrifuge tube and separated by centrifugation. Three biological replicates were each analyzed in triplicate. To extract intracellular metabolites, the resulting pellet was quenched and simultaneously extracted in 250 μL of ice-cold methanol and stored at −20 °C until analysis. For the extracellular metabolite analysis, three parts of supernatant were transferred to a microcentrifuge tube containing one part ice-cold methanol and mixed by vortexing. Two volumes of acetonitrile were added to the resulting mixture. All extracts were analyzed using UPLC/HRMS. All UPLC/HRMS analysis was done with an Agilent 1290 Infinity II system equipped with a multisampler, degasser, quaternary pump, and temperature controlled column compartment in tandem with an ESI and a Bruker Compact qTOF.

Chromatography for polar metabolites was performed with an Agilent InfinityLab Poroshell 120 HILIC-Z 2.1 × 150 mm × 2.7 µm PEEK-lined column and accompanying guard columns. Analysis was performed only in negative mode. The column and instrument were flushed with 0.5% phosphoric acid overnight in accordance with the Agilent Deactivator Additive protocol. The system was then equilibrated to the mobile phase conditions for data acquisition. Line A mobile phase was 10 mM ammonium acetate in water with 2.5 µM InfinityLab Deactivator Additive, adjusted to pH 9.0 with ammonium hydroxide. Line B was 10 mM ammonium acetate in 85:15 acetonitrile:water and 2.5 µM InfinityLab Deactivator Additive, adjusted to pH 9.0 with ammonium hydroxide. At the start of the run, the mobile phase consisted of 96% solvent B, which was maintained for the first 2 min. The %B was then reduced to 88% over the next 3.5 min and held steady for an additional 3 min. At the 9-min mark, the %B was further reduced to 86% and held steady for the next 5 min. At 17 min, %B was decreased to 82% for 3 min. %B was returned to 96% between 23 and 24.5 min, and held at 96% until the end of the run. The method included a 3 min post-run. The column was held at 50 °C. The flow rate was 0.25 mL/min and the injection volume was 3 µL. The autosampler was maintained at 4 °C over the analytical run. The MS was calibrated with sodium formate salt before the analytical run. Source parameters were as follows; end plate offset of 500 V, nebulizer pressure at 2.8 Bar, and capillary voltage of 2500 V. The dry gas flow rate was 6.0 l/min, and the dry temperature was set to 225 °C.

Chromatography for non-polar metabolites was done with an InfintityLab Poroshell 120 EC-C18,2.1 mm × 100 mm, 1.9 µm and the accompanying guard column. Analysis was performed only in negative mode. Line A was water with 5 mM ammonium formate and 0.01% FA, while line B was methanol with 0.01% FA. The gradient started at 65% line B, ran for 2 min, and then ramped to 77% B until 14 min. From 14 to 16.4 min, the method ramped to 95% B. From 16.4 to 20 min, the method equilibrated back to 65% B. A post-time was not included. The flow rate was 500 μL/min, and the column temperature was 50 °C. The multisampler was held at ambient temperature, and methanol was used for washing the needle in between injections. Source settings were an end plate offset of 500 V, a nebulizer pressure of 4.0 Bar, a dry temperature of 220 °C, a capillary voltage of 2500 V, and a dry gas flow rate of 10.0 l/min.

### Metabolomics data analysis

All chromatograms were visualized and processed using Bruker Data Analysis software or Bruker Metaboscape© software. Statistical analysis consisted of PCA and ANOVA. PCA was performed with no scaling algorithm, 1% cross validation, and 95.0 minimum explained variance. Datasets were then analyzed with ANOVA, where treatment group was the target variable and substrate (blank or pellet) was the limiting group. This produced a short list of significant compounds, which was cross validated with another ANOVA focusing on sample or blank as the target condition. Overlapping significant compounds were then examined visually and compounds that were strikingly higher in the CBD-fed cell pellets were chosen as the target compounds to identify in the study. Compound annotation was performed using MetFrag where MS1 data were searched for in KEGG, MetaCyc, LipidMaps, Pubchem, and Chebi databases. Where possible, MS2 spectra were then evaluated to probe for further support of the compound identity. Further compound elucidation was performed by screening the data for known cannabidiol MS artifacts and known cannabidiol microbial metabolites such as monohydroxylated CBD, dihydroxylated CBD, and *o*-glycosylated CBD, however, no metabolites were identified. Last, a pathway analysis was conducted using Metaboanalyst based on the significant compounds that we reported in this study.

### Knockout and rescue

Kanamycin resistant insertion cassettes were constructed using Gibson assembly^62^ into a pCfB2791 backbone (Addgene plasmid #63654). *URA3* was taken from pYTK074 from the Yeast Tool Kit (Addgene kit #1000000061) for deletion of *CIS1*. All strains were produced using the LiAc/single stranded carrier DNA/PEG method^63^ with linear insertion cassettes for targeted homology directed repair. All insertion cassettes were added in a quantity of 1 μg with equimolar amounts of 500 bp homology arms flanking either side of the insert. Insertion cassettes were amplified using primers with 50 bp overhang homologous to the homology arms. A diagram of the Δ*pdr5* insertion cassette is shown in Supplementary Information Fig. 64 and the GenBank file for the sequence is attached to this Supplementary Information in a separate document. After cell transformation, cells were grown in nonselecting rich media for at least 4 h and then plated to a selection media agar plate. Plates were monitored until colonies appeared and then colonies were screened using PCR at the locus of interest followed by Sanger sequencing. Continual growth monitoring with CBD treatment was performed as previously described.

### Reporting summary

Further information on research design is available in the Nature Research Reporting Summary linked to this article.

### Supplementary information

Supplementary Information

Reporting summary

Related Manuscript File

## Data Availability

Data for this experiment are available in the supplementary material of this article and all raw experimental data are available upon request. Some figures within were digitally retouched for ease of the reader’s interpretation without manipulation of information that is relevant to the interpretation of the figures, and originals are retained and available. Genome assembly is available from NCBI via accession code PRJNA1106478. Transcriptomic data (raw reads, sorted by barcode) are available from NCBI via accession code PRJNA1106505.
