# Supplementary material for: Integrated omics of Saccharomyces cerevisiae CENPK2-1C reveals pleiotropic drug resistance and lipidomic adaptations to cannabidiol
Source: NPJ Syst Biol Appl. 2024 May 31;10:63. doi: 10.1038/s41540-024-00382-0 (PMC11143246; doi:10.1038/s41540-024-00382-0)
Supplement: Supplementary file 1 — Supplementary Information [file 41540_2024_382_MOESM1_ESM.pdf]

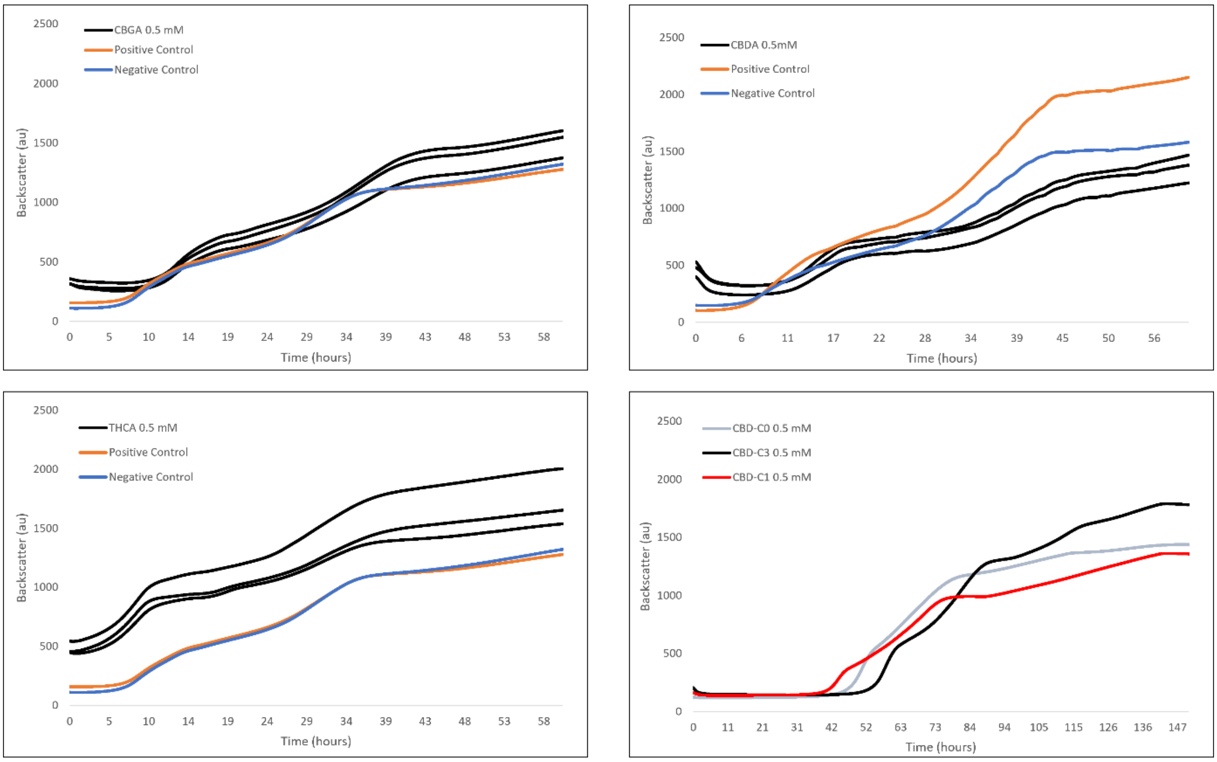

Supplementary Figure 1: Top left - CBGA was fed to a yeast cell culture at a concentration of 0.5 mM. at the time of inoculation, started at 0.1 ODU/mL. Bottom left- THCA was fed to a yeast cell culture at a concentration of 0.5 mM. at the time of inoculation, started at 0.1 ODU/mL. Top right- CBDA was fed to a yeast cell culture at a concentration of 0.5 mM. at the time of inoculation, started at 0.1 ODU/mL. Acidic cannabinoid each exerted a unique pattern of cell growth and adaptation. Bottom right- yeast were given cannabidivarin (CBD-C3), or cannabidiorcol (CBD-C1), and cannabidiresoricol.(CBD-C0). Short-alkyl chain cannabinoids suspended growth entirely until the cells overcame the influence of the cannabinoids.

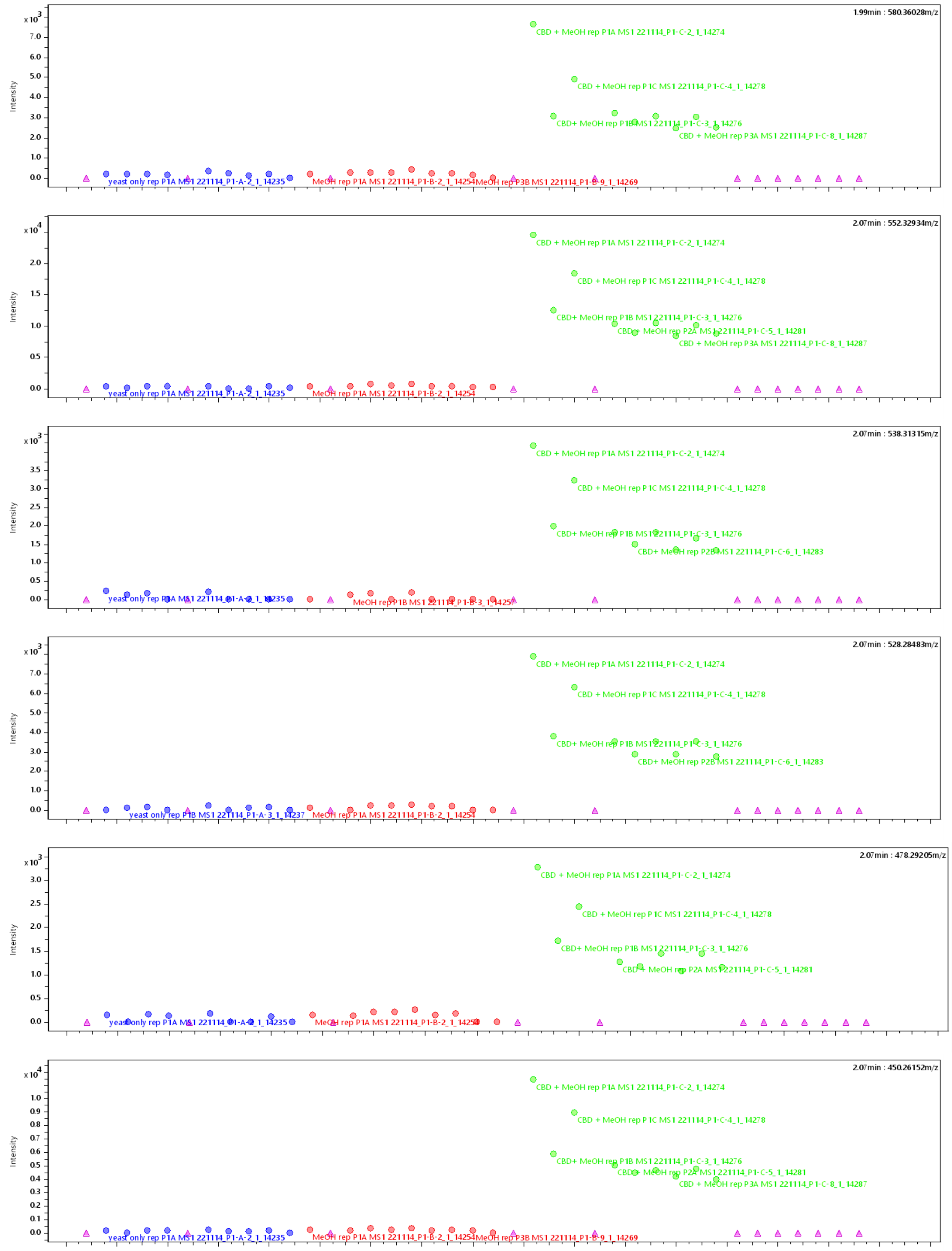

Supplementary Figure 2: The shared pattern of intensity across the spectra at 2.0 min at m/z 580.36028 (parent), 552.32934 (F1, confirmed in MetFrag), 538.31315 (F2, confirmed in MetFrag), 528.28483 (F3, not found in any database) and 478.29205 (F4, confirmed in MetFrag) led us to conclude that all of these spectra were related as a single compound. In spite of no collision energy, we submitted the smaller spectra as fragments in MetFrag and found database hits for 3 out of 4 spectra.

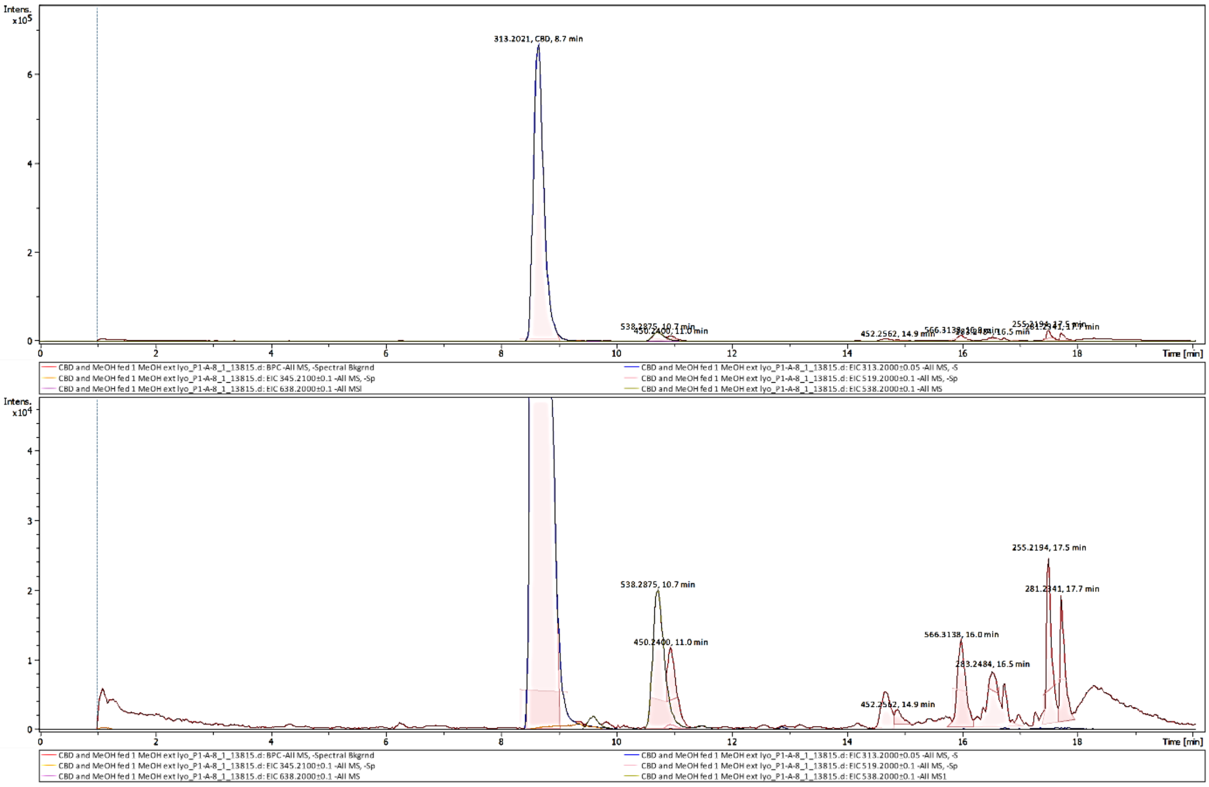

Supplementary Figure 3: HRMS Raw Chromatogram of CBD-fed cell pellet samples using reverse phase C18 chromatography. (Top) Overview of entire chromatogram. (Bottom) Zoomed in view to show information about smaller peaks. Labels are of the base peak in each integration followed by the retention time.

Supplementary Table 1: Nanoplot statistics from *S. cerevisiae* CENPK2-1C gDNA sequenced using SQK-LSK109 kit and R9.1.4 flow cell on an Oxford Nanopore MinION MK1C. Basecalliing was performed in Guppy using super accurate basecalling.

**number_of_reads**	477258
**number_of_bases**	5043072114.0
**median_read_length**	8554.0
**mean_read_length**	10566.8
**read_length_stdev**	8256.7
**n50**	15469.0
**mean_qual**	15.5
**median_qual**	15.8
**longest_read_(with_Q):1**	101896 (16.4)
**longest_read_(with_Q):2**	96770 (10.4)
**longest_read_(with_Q):3**	91864 (17.8)
**longest_read_(with_Q):4**	477258
**longest_read_(with_Q):5**	5043072114.0
**highest_Q_read_(with_length):1**	8554.0
**highest_Q_read_(with_length):2**	10566.8
**highest_Q_read_(with_length):3**	8256.7
**highest_Q_read_(with_length):4**	23.3 (1074)
**highest_Q_read_(with_length):5**	23.1 (25994)
**highest_Q_read_(with_length):4**	23.3 (1074)
**highest_Q_read_(with_length):5**	23.1 (25994)
**Reads >Q5:**	477258 (100.0%) 5043.1Mb
**Reads >Q7:**	477258 (100.0%) 5043.1Mb
**Reads >Q10:**	477247 (100.0%) 5043.1Mb
**Reads >Q12:**	426945 (89.5%) 4480.9Mb
**Reads >Q15:**	290826 (60.9%) 3079.6Mb

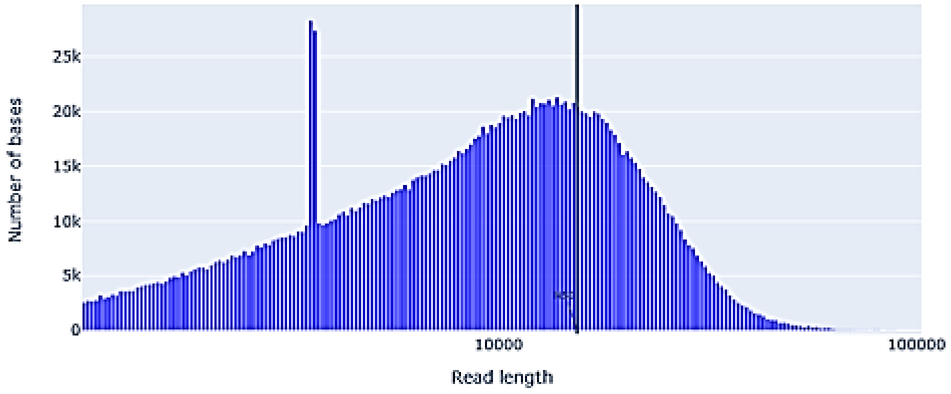

Supplementary Figure 4: Nanoplot weighted histogram from *S. cerevisiae* CENPK2-1C gDNA sequenced using SQK-LSK109 kit and R9.1.4 flow cell on an Oxford Nanopore MinION MK1C. Basecalliing was performed in Guppy using super accurate basecalling. The N50 is shown by the black bar.

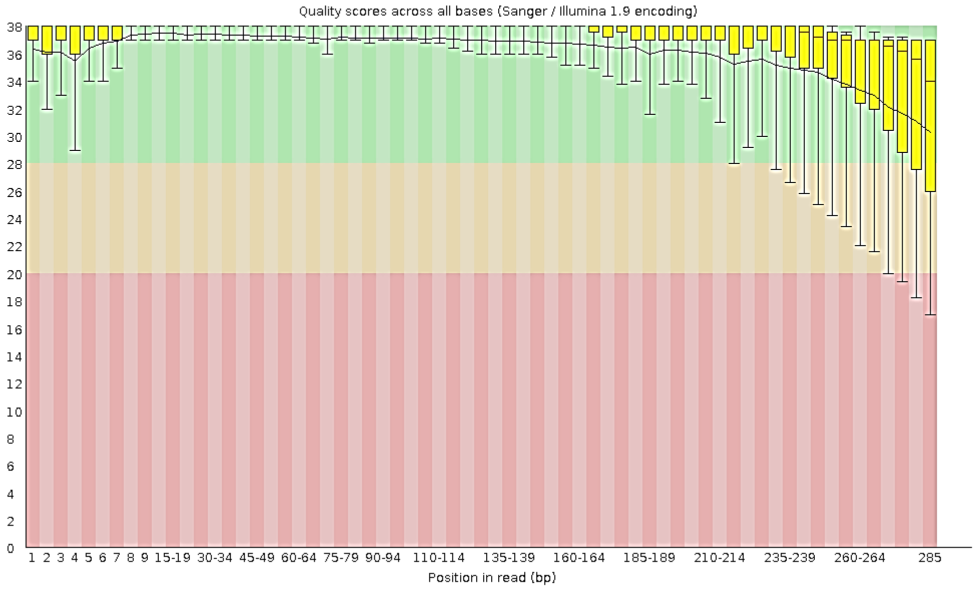

Supplementary Figure 5: FastQC Quality scores of paired Illumina reads of *S. cerevisiae* CENPK2-1C gDNA after trimming using Trimmomatic.

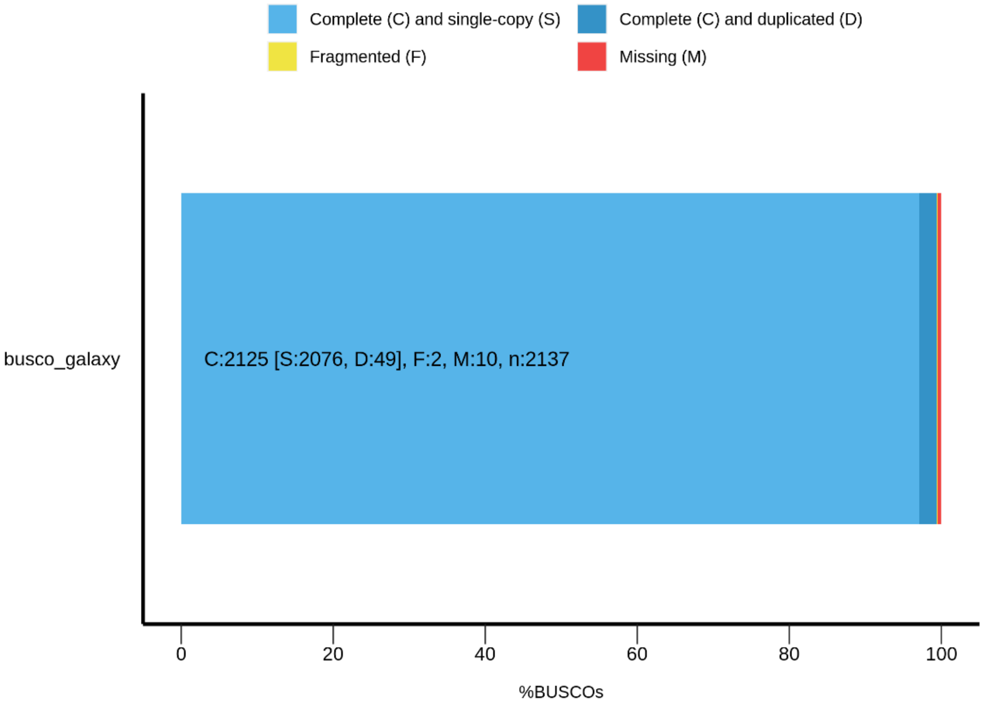

Supplementary Figure 6: BUSCO Assessment Results after assembling *S. cerevisiae* CENPK2-1C genome on a total of 28 contigs with an N50 of 800.5 Kbp and a genome size of 11.98 Mbp. Augustus was used as the gene predictor. This BUSCO gave a completeness score of 99.4% and found 2125 complete BUSCOs, 2076 complete and single-copy BUSCOs, 49 complete and duplicated BUSCOs, 2 fragmented BUSCOs, 10 missing BUSCOs, and searched a total of 2137 BUSCOs. Assembly statistics were; 28 scaffolds, 83 contigs, length of 11,994,286, 0.005% gaps, 800KB scaffold N50, and 309 contig N50.

Supplementary Table 2: Short summary of cDNA libraries used in transcriptomics

Group	Barcode	Number of Reads	Included in DESeq2
Positive control	BC01	92371	Included
Positive control	BC02	633012	Included
Positive control	BC03	2379021	Included
0.5 mM CBD fed	BC04	104495	Included
0.5 mM CBD fed	BC05	1496390	Included
0.5 mM CBD fed	BC06	8920	Excluded
Logarithmic phase control	None	3104404	Included

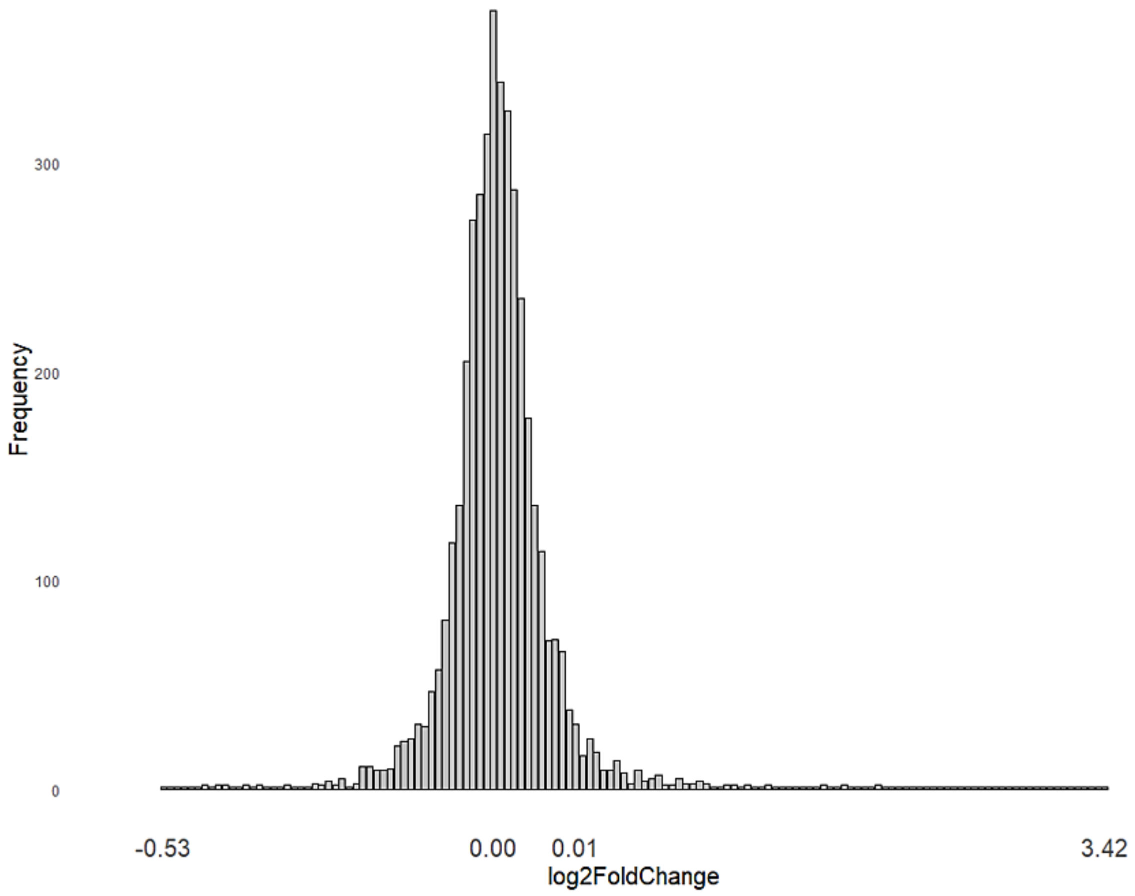

Supplementary Figure 7: Binned Histogram of DESeq2-produced log2 fold change values between CBD-fed, MeOH-fed, and mid-logarithmic phase *S. cerevisiae* CENPK2-1C cDNA libraries sequenced with Oxford Nanopore MK1C using SQK-PCB109 and a R9.1.4 flow cell. The mean of the data was 0.006. The x-axis was binned to show the distribution of the data, where a continuous x-axis would have shown virtually all data at the 0.0 point without any clarity about the shape of the data. The lowest value was -0.53 and the highest was 3.42.

Supplementary Table 3: Nanoplot statistics from barcoded cDNA from *S. cerevisiae* CENPK2-1C RNA sequenced using SQK-PCB109 kit and R9.1.4 flow cell on an Oxford Nanopore MinION MK1C. Basecalliing was performed in Guppy using super accurate basecalling.

	MeOH treated positive control			CBD fed experimental group			
Metrics	BC01	BC02	BC03	BC04	BC05	BC06	Mid-log phase control No barcode
number_of_reads	92371	633012	2379021	104495	1496390	8920	3104404
number_of_bases	66284106	360685047	843216336	69189946	900399094	7145919	1270185489
median_read_length	569	398	294	562	508	639.5	305
mean_read_length	717.6	569.8	354.4	662.1	601.7	801.1	409.2
read_length_stdev	536.9	457.7	191	438.4	391.3	565.5	299.4
n50	919	712	351	756	718	1024	435
mean_qual	12.7	12.4	11.9	12.6	12.5	12.7	12
median_qual	12.5	12.2	11.7	12.5	12.3	12.6	11.8
longest_read_(with_Q):1	11382 (14.5)	9599 (13.8)	16872 (11.0)	5413 (11.4)	10675 (16.1)	5718 (11.2)	16872 (11.0)
longest_read_(with_Q):2	6067 (14.1)	6026 (15.5)	5740 (10.3)	4574 (15.1)	5358 (12.6)	5220 (16.4)	11382 (14.5)
longest_read_(with_Q):3	5639 (15.9)	5673 (11.5)	4847 (12.8)	4280 (14.0)	5156 (16.0)	5217 (15.7)	9599 (13.8)
longest_read_(with_Q):4	5590 (12.7)	5665 (10.7)	4461 (14.6)	4252 (11.2)	5045 (10.2)	4898 (16.3)	6067 (14.1)
longest_read_(with_Q):5	5534 (13.8)	5642 (11.3)	4261 (11.9)	4126 (12.5)	5044 (13.3)	4780 (13.5)	6026 (15.5)
highest_Q_read_(with_length):1	19.7 (475)	25.1 (203)	22.4 (210)	20.6 (2119)	23.0 (391)	18.8 (1806)	25.1 (203)
highest_Q_read_(with_length):2	19.5 (3152)	21.7 (227)	22.3 (174)	20.1 (341)	22.9 (215)	18.1 (1983)	22.4 (210)
highest_Q_read_(with_length):3	19.4 (3076)	21.5 (620)	22.0 (155)	19.6 (1435)	22.8 (394)	18.1 (1609)	22.3 (174)
highest_Q_read_(with_length):4	18.9 (1370)	21.4 (369)	21.6 (226)	19.3 (589)	22.5 (223)	18.1 (2174)	22.0 (155)
highest_Q_read_(with_length):5	18.9 (1530)	21.1 (232)	21.6 (245)	19.3 (1667)	22.4 (235)	17.9 (1653)	21.7 (227)
Reads >Q5:	92371 (100.0%) 66.3Mb	633012 (100.0%) 360.7Mb	2379021 (100.0%) 843.2Mb	104495 (100.0%) 69.2Mb	1496390 (100.0%) 900.4Mb	8920 (100.0%) 7.1Mb	3104404 (100.0%) 1270.2Mb
Reads >Q7:	92371 (100.0%) 66.3Mb	633012 (100.0%) 360.7Mb	2379021 (100.0%) 843.2Mb	104495 (100.0%) 69.2Mb	1496390 (100.0%) 900.4Mb	8920 (100.0%) 7.1Mb	3104404 (100.0%) 1270.2Mb
Reads >Q10:	92370 (100.0%) 66.3Mb	632999 (100.0%) 360.7Mb	2379006 (100.0%) 843.2Mb	104487 (100.0%) 69.2Mb	1496338 (100.0%) 900.3Mb	8919 (100.0%) 7.1Mb	3104375 (100.0%) 1270.2Mb
Reads >Q12:	56777 (61.5%) 48.0Mb	340963 (53.9%) 243.2Mb	973028 (40.9%) 409.7Mb	63479 (60.7%) 49.0Mb	864528 (57.8%) 616.1Mb	5547 (62.2%) 5.2Mb	1370768 (44.2%) 701.0Mb
Reads >Q15:	8389 (9.1%) 11.0Mb	40703 (6.4%) 49.5Mb	30510 (1.3%) 21.6Mb	8157 (7.8%) 9.6Mb	96675 (6.5%) 103.0Mb	779 (8.7%) 1.1Mb	79602 (2.6%) 82.1Mb

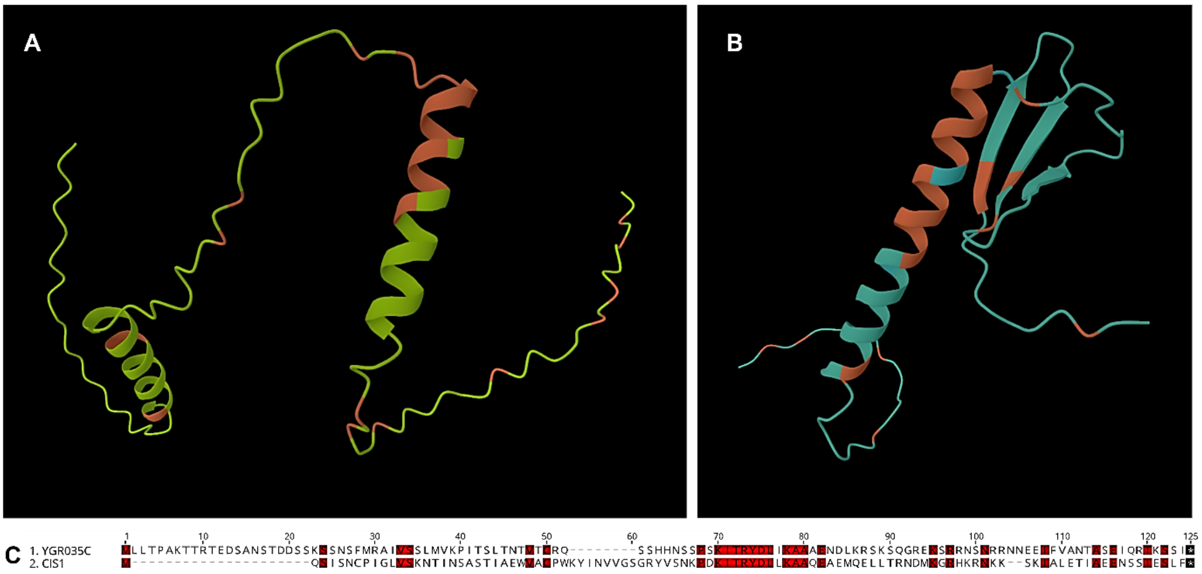

Supplementary Figure 8: Alpha-fold structures in 3D and protein sequence alignment of Ygr035c and Cis1. Ygr035c is shown in A. Cis1 is shown in B. A protein sequence alignment is shown in C. In A and B, conserved residues are shown in red. Although these genes are paralogous, they share only 21.6% shared sequence identity at the amino acid level, and they are structurally divergent. The function of these proteins is not yet clear, but we predict that they function in different aspects of the pleiotropic drug resistance response.

Supplementary Table 4: WoLFPSORT prediction scores for each subcellular compartment for Ygr035c and Cis1. The nucleus was the highest scoring subcellular compartment for Ygr035c and the mitochondria was the highest scoring subcellular compartment for Cis1.

Subcellular compartment	Ygr035c	Cis1
Mitochondria	2	**16.5**
Mitochondrial Nucleus	N/A	12.333
Nucleus	**18.5**	7
Nuclear Cytosol	14	4.833
Peroxisome	N/A	2
Cytosol	6.5	1.5

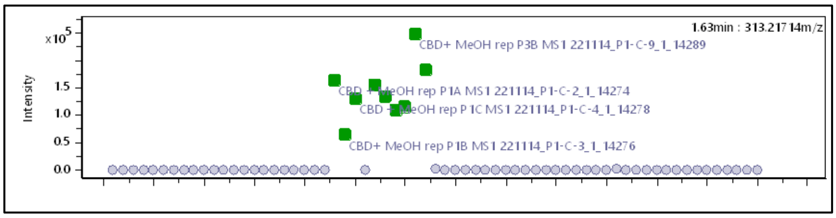

Supplementary Figure 9: Intensity plot of RT 1.63min 313.21714 m/z (Cannabidiol)

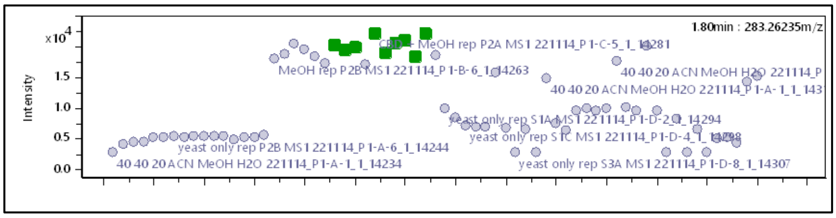

Supplementary Figure 10: Intensity plot of RT 1.80min 283.26235 m/z (Stearic acid)

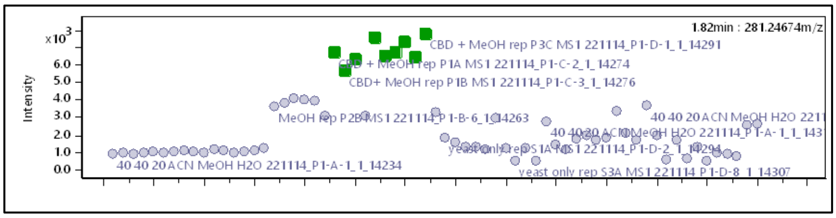

Supplementary Figure 11: Intensity plot of RT 1.82min 281.24674 m/z (2-Hydroxystearic acid)

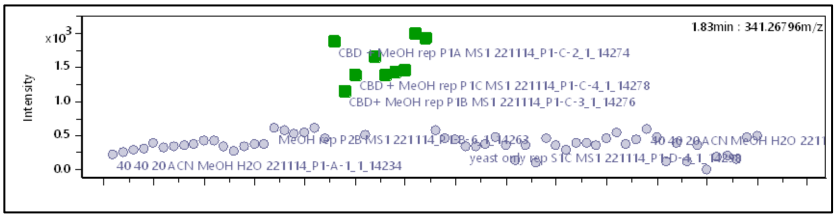

Supplementary Figure 12: Intensity plot of RT 1.83min 341.26796 m/z (Cannabidiol-o-methyl-o-methyl, artifact)

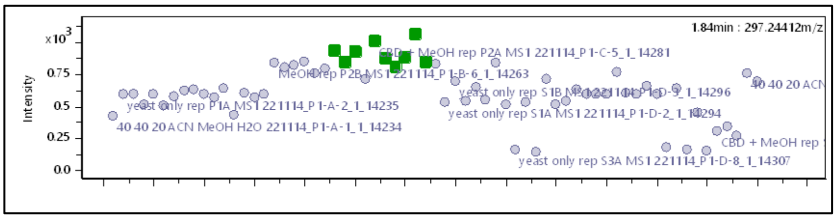

Supplementary Figure 13: Intensity plot of RT 1.84min 297.24412 m/z (9-Hydroxy-12-octadecenoic acid)

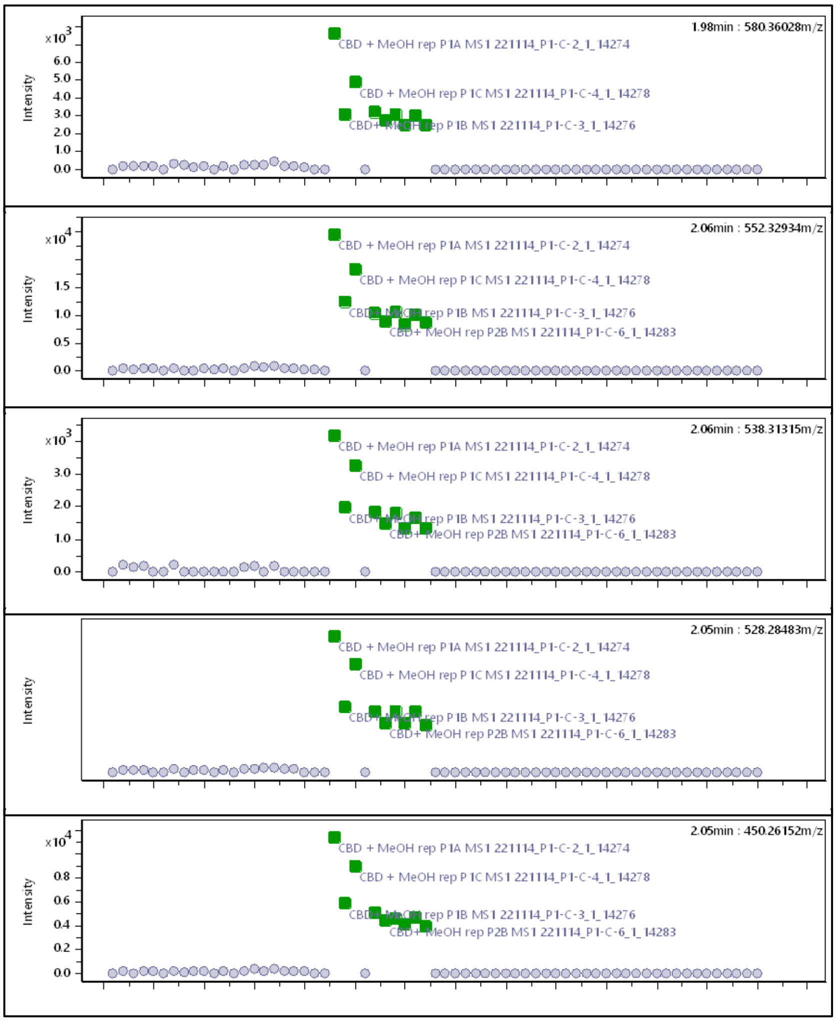

Supplementary Figure 14: Intensity plot of RT 1.98-2.05min 580.36028 m/z [M-], 552.32934 m/z [F1], 538.31315 m/z [F2], 528.28483 m/z [F3], 450.23152 m/z [F4] (1-docosanoyl-glycero-3-phosphoserine)

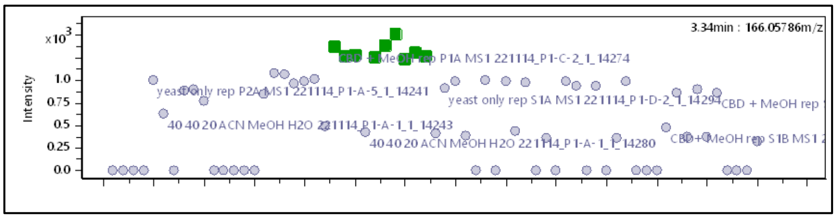

Supplementary Figure 15: Intensity plot of RT 3.34min 166.05786 m/z (not yet identified)

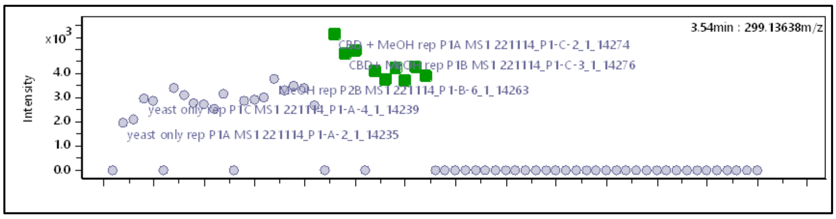

Supplementary Figure 16: Intensity plot of RT 3.54min 299.13638 m/z (not yet identified)

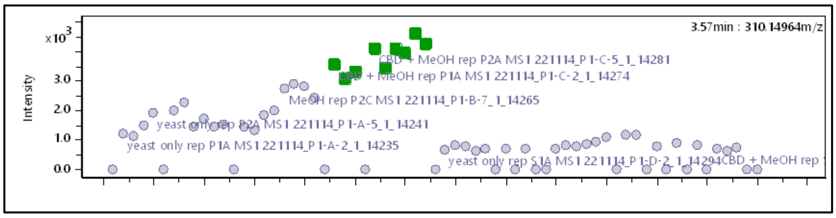

Supplementary Figure 17: Intensity plot of RT 3.57min 310.14964 m/z (not yet identified)

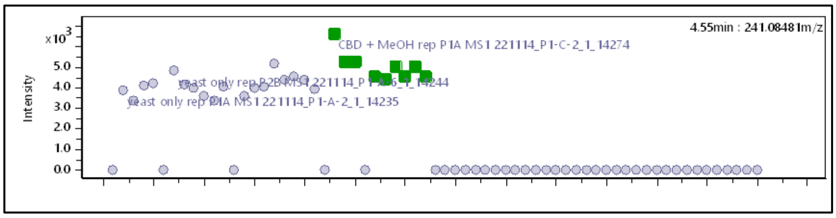

Supplementary Figure 18: Intensity plot of RT 4.55min 241.08487 m/z (Thymidine)

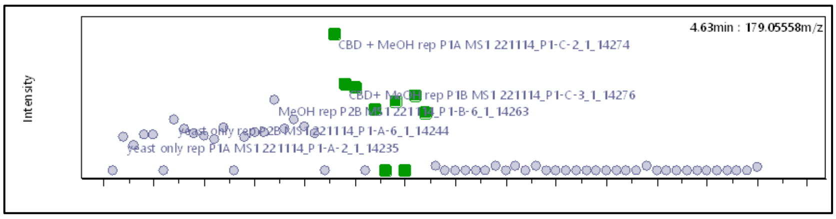

Supplementary Figure 19: Intensity plot of RT 4.63min 179.05558 m/z (D-glucose)

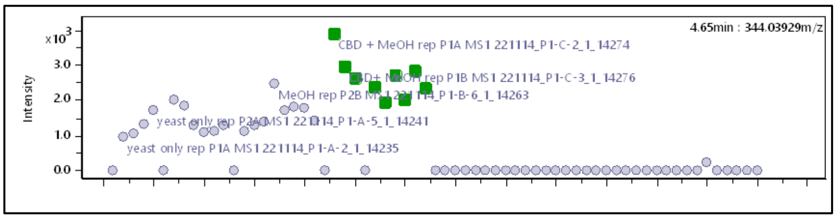

Supplementary Figure 20: Intensity plot of RT 4.65min 344.03929 m/z (Nucleotide monophosphate e.g. adenosine 2'-phosphate2- or AMP or dGMP)

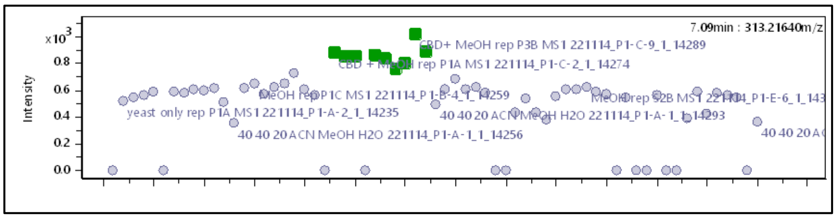

Supplementary Figure 21: Intensity plot of RT 7.09min 313.21640 m/z (Cannabidiol in tandem with another compound)

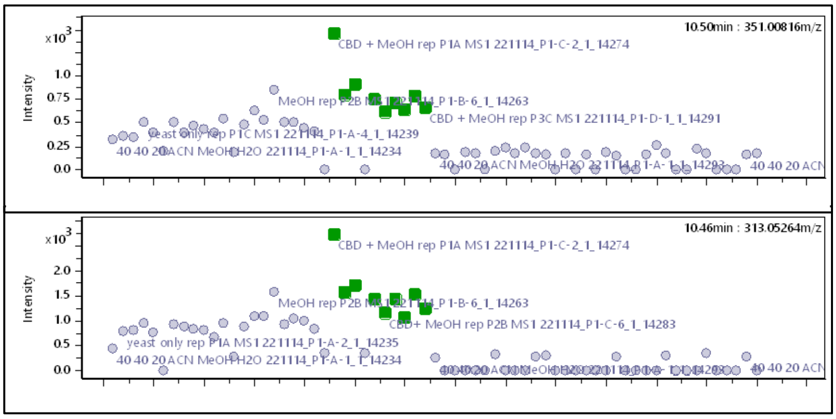

Supplementary Figure 22: Intensity plot of RT 10.46-10.50min 351.00819 m/z [M-], 313.05264 m/z [F1] (not yet identified)

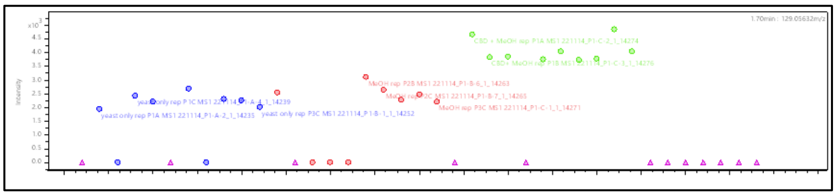

Supplementary Figure 23: Intensity plot of RT 1.70min 129.05632 m/z [M-] (not yet identified). Purple triangle = solvent blank, blue circle = negative control cell pellet, red circle = positive control cell pellet, green circle = CBD-fed cell pellet

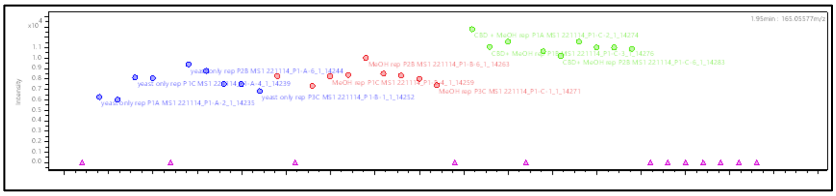

Supplementary Figure 24: Intensity plot of RT 1.95min 165.05577 m/z [M-] (not yet identified). Purple triangle = solvent blank, blue circle = negative control cell pellet, red circle = positive control cell pellet, green circle = CBD-fed cell pellet

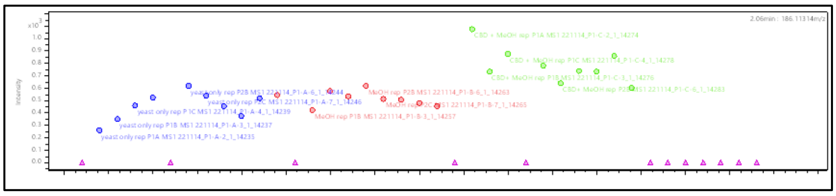

Supplementary Figure 25: Intensity plot of RT 2.06min 186.11314 m/z [M-] (8-Amino-7-oxononanoate). Purple triangle = solvent blank, blue circle = negative control cell pellet, red circle = positive control cell pellet, green circle = CBD-fed cell pellet

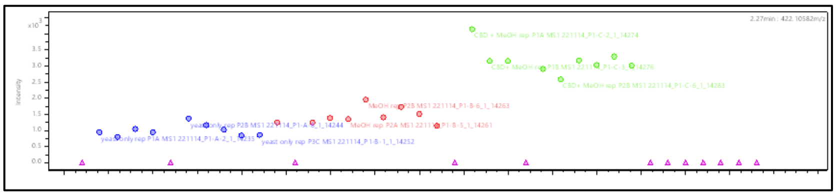

Supplementary Figure 26: Intensity plot of RT 2.27min 422.10582 m/z [M-] (not yet identified). Purple triangle = solvent blank, blue circle = negative control cell pellet, red circle = positive control cell pellet, green circle = CBD-fed cell pellet

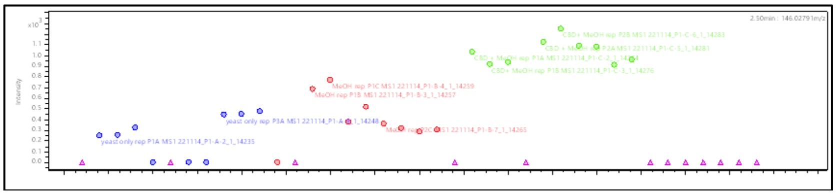

Supplementary Figure 27: Intensity plot of RT 2.50min 146.02791 m/z [M-] (Thiomorpholine 3-carboxylate). Purple triangle = solvent blank, blue circle = negative control cell pellet, red circle = positive control cell pellet, green circle = CBD-fed cell pellet

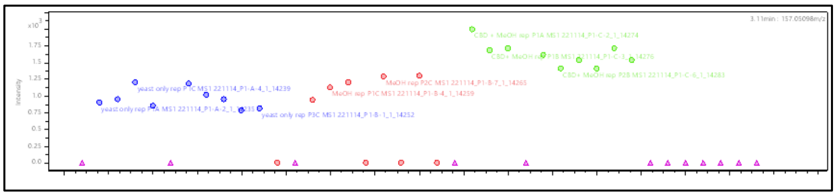

Supplementary Figure 28: Intensity plot of RT 3.11min 157.05098 m/z [M-] (not yet identified). Purple triangle = solvent blank, blue circle = negative control cell pellet, red circle = positive control cell pellet, green circle = CBD-fed cell pellet

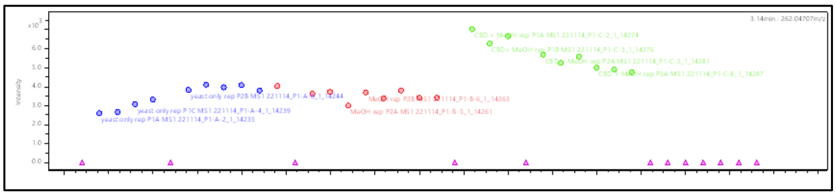

Supplementary Figure 29: Intensity plot of RT 3.14min 262.40707 m/z [M-] (not yet identified). Purple triangle = solvent blank, blue circle = negative control cell pellet, red circle = positive control cell pellet, green circle = CBD-fed cell pellet

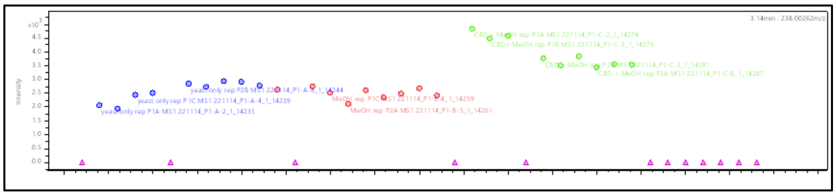

Supplementary Figure 29: Intensity plot of RT 3.14min 238.00262 m/z [M-] (not yet identified). Purple triangle = solvent blank, blue circle = negative control cell pellet, red circle = positive control cell pellet, green circle = CBD-fed cell pellet

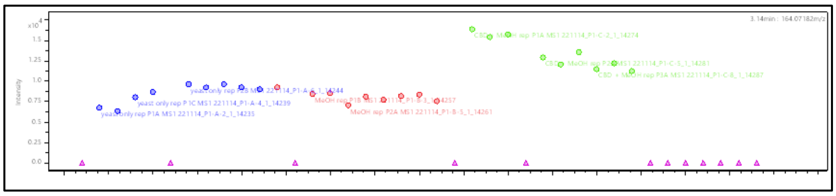

Supplementary Figure 30: Intensity plot of RT 3.14min 164.07182 m/z [M-] (L-Phenylalanine). Purple triangle = solvent blank, blue circle = negative control cell pellet, red circle = positive control cell pellet, green circle = CBD-fed cell pellet

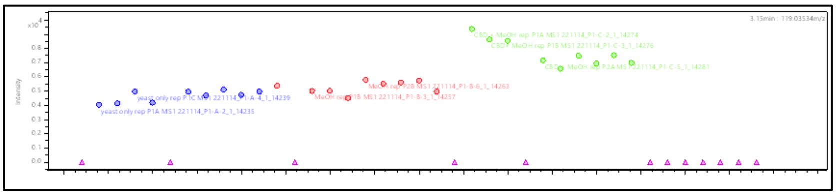

Supplementary Figure 31: Intensity plot of RT 3.15min 119.03534 m/z [M-] (D-Erythrose). Purple triangle = solvent blank, blue circle = negative control cell pellet, red circle = positive control cell pellet, green circle = CBD-fed cell pellet

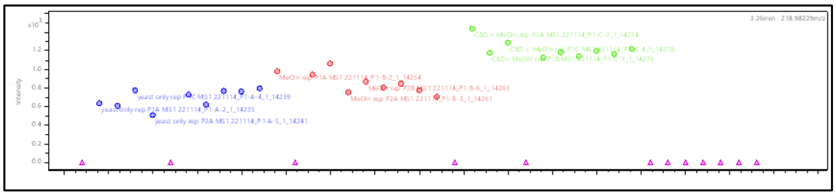

Supplementary Figure 32: Intensity plot of RT 3.26min 218.98229 m/z [M-] (not yet identified). Purple triangle = solvent blank, blue circle = negative control cell pellet, red circle = positive control cell pellet, green circle = CBD-fed cell pellet

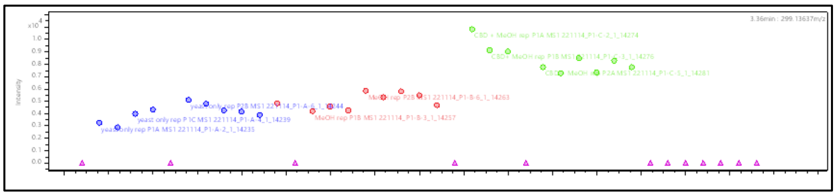

Supplementary Figure 33: Intensity plot of RT 3.36min 299.13637 m/z [M-] (not yet identified). Purple triangle = solvent blank, blue circle = negative control cell pellet, red circle = positive control cell pellet, green circle = CBD-fed cell pellet

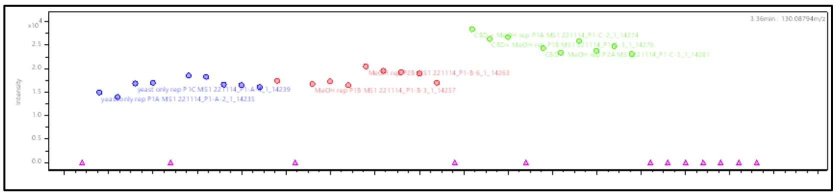

Supplementary Figure 34: Intensity plot of RT 3.36min 130.08794 m/z [M-] (L-Leucine). Purple triangle = solvent blank, blue circle = negative control cell pellet, red circle = positive control cell pellet, green circle = CBD-fed cell pellet

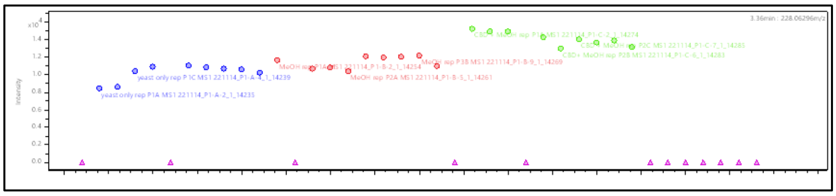

Supplementary Figure 35: Intensity plot of RT 3.36min 228.06296 m/z [M-] (not yet identified). Purple triangle = solvent blank, blue circle = negative control cell pellet, red circle = positive control cell pellet, green circle = CBD-fed cell pellet

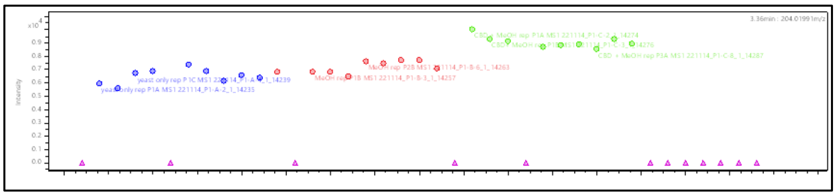

Supplementary Figure 36: Intensity plot of RT 3.36min 204.01991 m/z [M-] (not yet identified). Purple triangle = solvent blank, blue circle = negative control cell pellet, red circle = positive control cell pellet, green circle = CBD-fed cell pellet

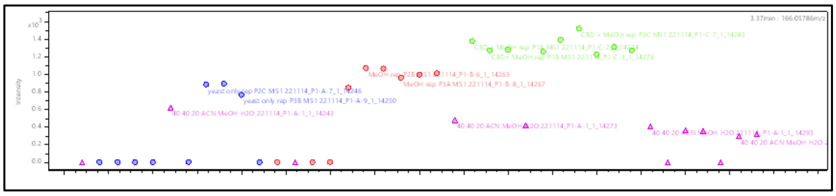

Supplementary Figure 37: Intensity plot of RT 3.37min 166.05786 m/z [M-] (not yet identified). Purple triangle = solvent blank, blue circle = negative control cell pellet, red circle = positive control cell pellet, green circle = CBD-fed cell pellet

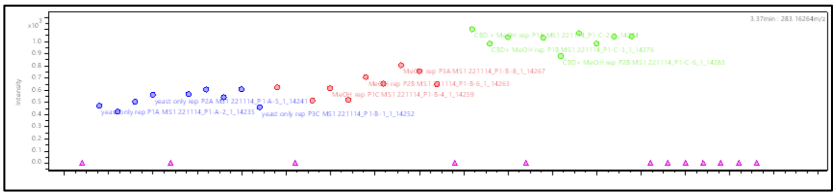

Supplementary Figure 38: Intensity plot of RT 3.37min 283.16264 m/z [M-] (not yet identified). Purple triangle = solvent blank, blue circle = negative control cell pellet, red circle = positive control cell pellet, green circle = CBD-fed cell pellet

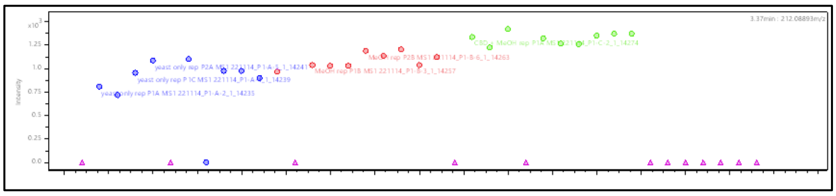

Supplementary Figure 39: Intensity plot of RT 3.37min 212.08893 m/z [M-] (not yet identified). Purple triangle = solvent blank, blue circle = negative control cell pellet, red circle = positive control cell pellet, green circle = CBD-fed cell pellet

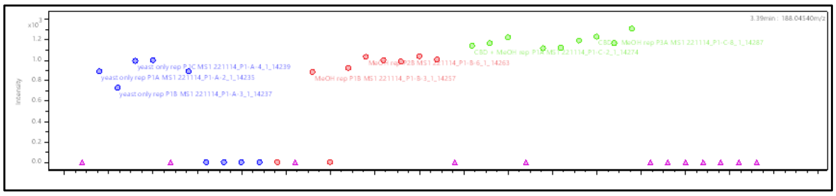

Supplementary Figure 40: Intensity plot of RT 3.39min 188.04540 m/z [M-] (not yet identified). Purple triangle = solvent blank, blue circle = negative control cell pellet, red circle = positive control cell pellet, green circle = CBD-fed cell pellet

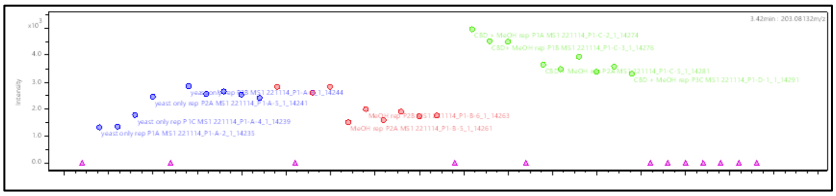

Supplementary Figure 41: Intensity plot of RT 3.42min 203.08132 m/z [M-] (L-Tryptophan). Purple triangle = solvent blank, blue circle = negative control cell pellet, red circle = positive control cell pellet, green circle = CBD-fed cell pellet

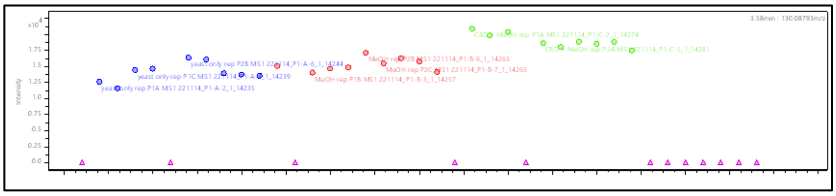

Supplementary Figure 42: Intensity plot of RT 3.58min 130.08793 m/z [M-] (L-Isoleucine). Purple triangle = solvent blank, blue circle = negative control cell pellet, red circle = positive control cell pellet, green circle = CBD-fed cell pellet

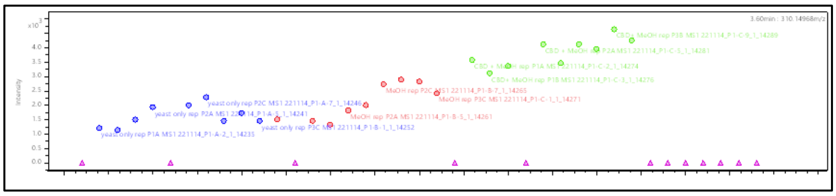

Supplementary Figure 43: Intensity plot of RT 3.60min 310.14968 m/z [M-] (not yet identified). Purple triangle = solvent blank, blue circle = negative control cell pellet, red circle = positive control cell pellet, green circle = CBD-fed cell pellet

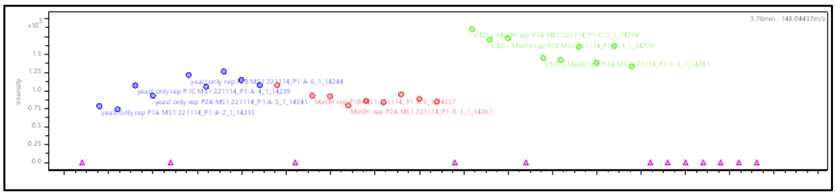

Supplementary Figure 44: Intensity plot of RT 3.76min 148.04437 m/z [M-] (L-Methionine). Purple triangle = solvent blank, blue circle = negative control cell pellet, red circle = positive control cell pellet, green circle = CBD-fed cell pellet

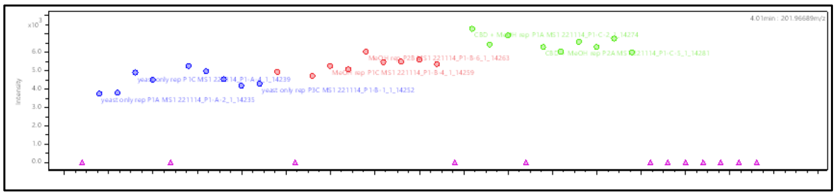

Supplementary Figure 45: Intensity plot of RT 4.01min 201.96689 m/z [M-] (not yet identified). Purple triangle = solvent blank, blue circle = negative control cell pellet, red circle = positive control cell pellet, green circle = CBD-fed cell pellet

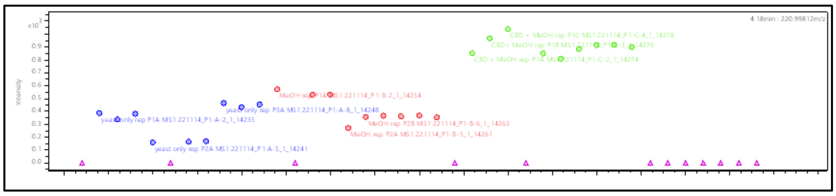

Supplementary Figure 46: Intensity plot of RT 4.18min 220.99812 m/z [M-2] (not yet identified). Purple triangle = solvent blank, blue circle = negative control cell pellet, red circle = positive control cell pellet, green circle = CBD-fed cell pellet

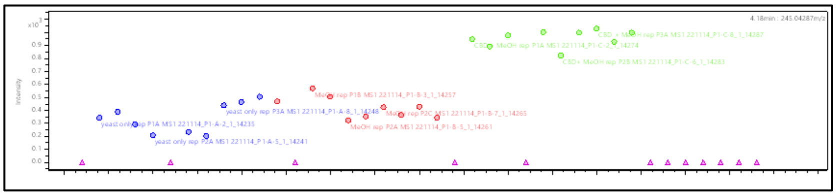

Supplementary Figure 47: Intensity plot of RT 4.18min 245.04287 m/z [M-] (glyercophosphoglycerol). Purple triangle = solvent blank, blue circle = negative control cell pellet, red circle = positive control cell pellet, green circle = CBD-fed cell pellet

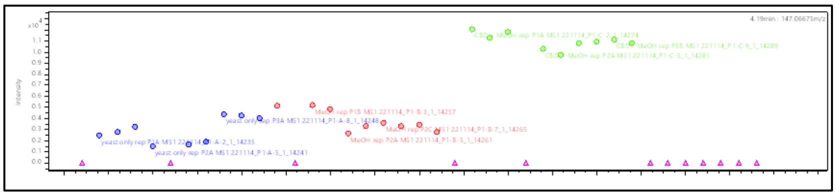

Supplementary Figure 48: Intensity plot of RT 4.19min 147.06675 m/z [M-] (not yet identified). Purple triangle = solvent blank, blue circle = negative control cell pellet, red circle = positive control cell pellet, green circle = CBD-fed cell pellet

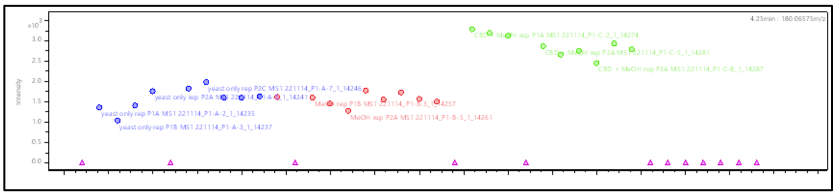

Supplementary Figure 49: Intensity plot of RT 4.25min 180.06575 m/z [M-] (L-Tyrosine). Purple triangle = solvent blank, blue circle = negative control cell pellet, red circle = positive control cell pellet, green circle = CBD-fed cell pellet

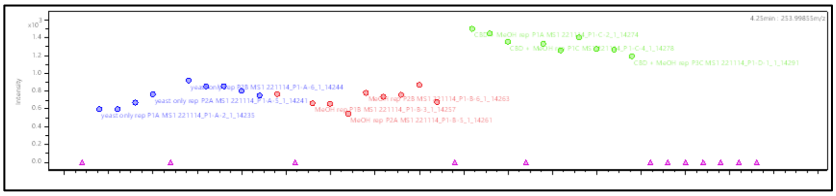

Supplementary Figure 50: Intensity plot of RT 4.25min 253.99855 m/z [M-] (not yet identified). Purple triangle = solvent blank, blue circle = negative control cell pellet, red circle = positive control cell pellet, green circle = CBD-fed cell pellet

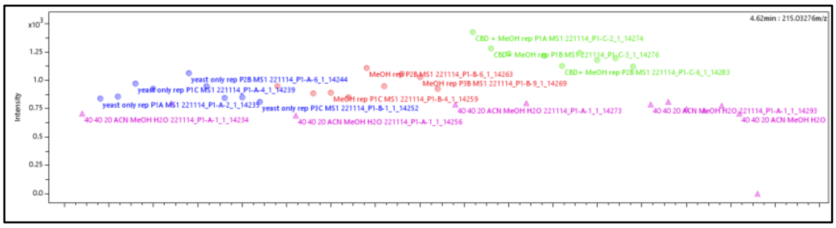

Supplementary Figure 51: Intensity plot of RT 4.62min 215.03276 m/z [M-] (not yet identified). Purple triangle = solvent blank, blue circle = negative control cell pellet, red circle = positive control cell pellet, green circle = CBD-fed cell pellet

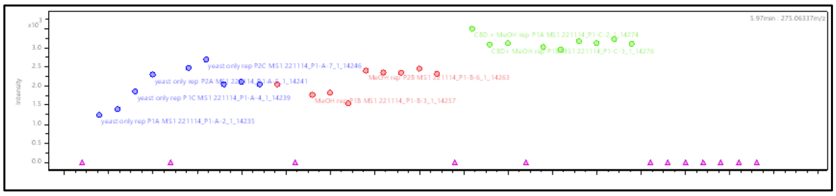

Supplementary Figure 52: Intensity plot of RT 5.97min 275.06337 m/z [M-] (not yet identified). Purple triangle = solvent blank, blue circle = negative control cell pellet, red circle = positive control cell pellet, green circle = CBD-fed cell pellet

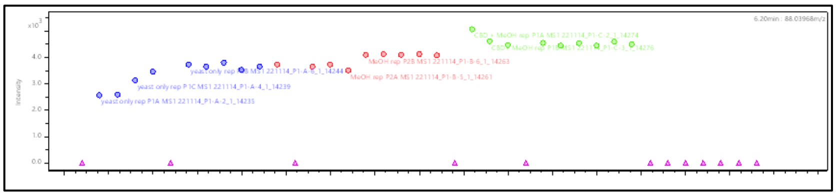

Supplementary Figure 53: Intensity plot of RT 6.20min 88.03968 m/z [M-] (L-Alanine). Purple triangle = solvent blank, blue circle = negative control cell pellet, red circle = positive control cell pellet, green circle = CBD-fed cell pellet

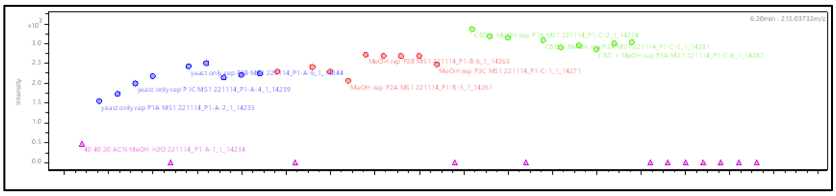

Supplementary Figure 54: Intensity plot of RT 6.20min 215.03732 m/z [M-] (not yet identified). Purple triangle = solvent blank, blue circle = negative control cell pellet, red circle = positive control cell pellet, green circle = CBD-fed cell pellet

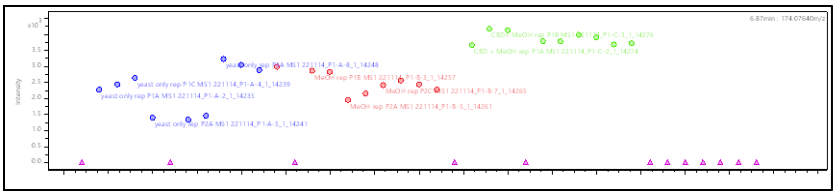

Supplementary Figure 55: Intensity plot of RT 6.87min 174.07640 m/z [M-] (not yet identified). Purple triangle = solvent blank, blue circle = negative control cell pellet, red circle = positive control cell pellet, green circle = CBD-fed cell pellet

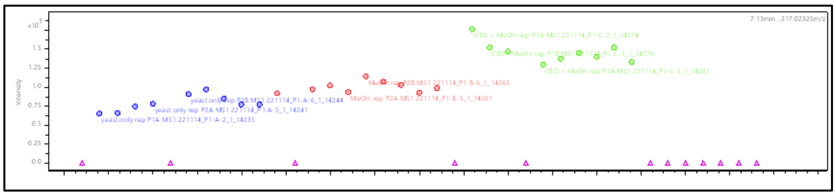

Supplementary Figure 56: Intensity plot of RT 7.13min 217.02325 m/z [M-] (not yet identified). Purple triangle = solvent blank, blue circle = negative control cell pellet, red circle = positive control cell pellet, green circle = CBD-fed cell pellet

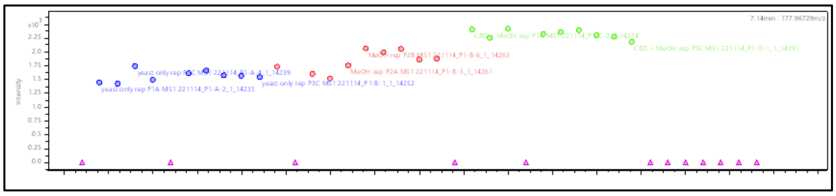

Supplementary Figure 57: Intensity plot of RT 7.14min 177.96729 m/z [M-] (not yet identified). Purple triangle = solvent blank, blue circle = negative control cell pellet, red circle = positive control cell pellet, green circle = CBD-fed cell pellet

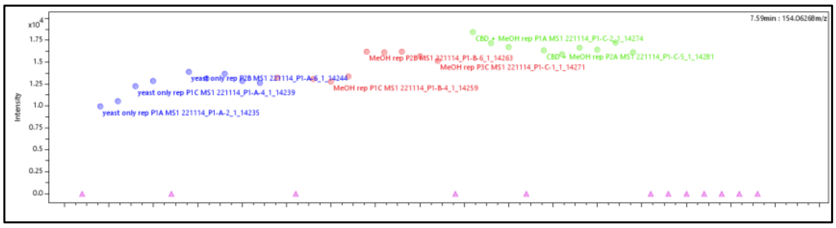

Supplementary Figure 58: Intensity plot of RT 7.59min 154.06268 m/z [M-] (L-Histidine). Purple triangle = solvent blank, blue circle = negative control cell pellet, red circle = positive control cell pellet, green circle = CBD-fed cell pellet

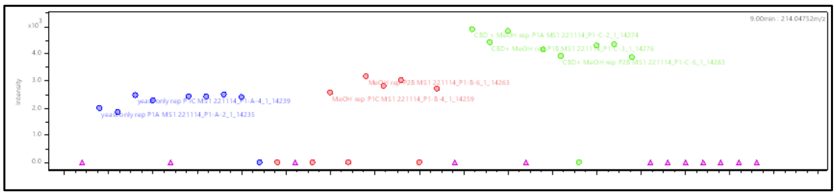

Supplementary Figure 59: Intensity plot of RT 9.00min 214.04752 m/z [M-] (sn-Glycero-3-phosphoethanolamine). Purple triangle = solvent blank, blue circle = negative control cell pellet, red circle = positive control cell pellet, green circle = CBD-fed cell pellet

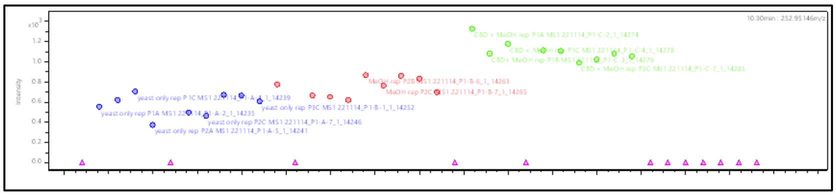

Supplementary Figure 60: Intensity plot of RT 10.30min 252.95146 m/z [M-] (not yet identified). Purple triangle = solvent blank, blue circle = negative control cell pellet, red circle = positive control cell pellet, green circle = CBD-fed cell pellet

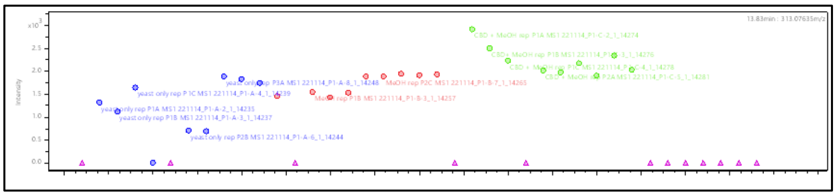

Supplementary Figure 61: Intensity plot of RT 13.83min 313.07635 m/z [M-] (not yet identified). Purple triangle = solvent blank, blue circle = negative control cell pellet, red circle = positive control cell pellet, green circle = CBD-fed cell pellet

Supplementary Table 5: Metabolanalyst Pathway Analysis results, input list; Stearic acid, 2R-hydroxy-stearic acid, Thymidine, D-Glucose, 3'-AMP,8-Amino-7-oxononanoate, Thiomorpholine 3-carboxylate, L-Phenylalanine ,Erythrose, L-Leucine, L-Tryptophan, L-Isoleucine, L-Methionine, sn-glycero-3-phosphoethanolamine, L-Tyrosine, L-Alanine, L-Histidine

	Total	Expected	Hits	Raw p	(-)LOG10(p)	Holm adjust	FDR	Impact
Aminoacyl-tRNA biosynthesis	46	0.62585	8	1.25E-08	7.9018	9.15E-07	9.15E-07	0
Phenylalanine, tyrosine and tryptophan biosynthesis	21	0.28571	3	0.002234	2.651	0.16083	0.081533	0.02144
Valine, leucine and isoleucine degradation	18	0.2449	2	0.023016	1.638	1	0.41101	0
Ubiquinone and other terpenoid-quinone biosynthesis	2	0.027211	1	0.027041	1.568	1	0.41101	0
Valine, leucine and isoleucine biosynthesis	20	0.27211	2	0.028151	1.5505	1	0.41101	0
Phenylalanine metabolism	7	0.095238	1	0.091738	1.0375	1	1	0
Biotin metabolism	13	0.17687	1	0.16416	0.78474	1	1	0
Tyrosine metabolism	15	0.20408	1	0.1871	0.72793	1	1	0
Histidine metabolism	18	0.2449	1	0.22042	0.65674	1	1	0
Alanine, aspartate and glutamate metabolism	22	0.29932	1	0.26291	0.58019	1	1	0
Biosynthesis of unsaturated fatty acids	23	0.31293	1	0.2732	0.56352	1	1	0
Glycolysis / Gluconeogenesis	24	0.32653	1	0.28335	0.54768	1	1	4.00E-04
Tryptophan metabolism	30	0.40816	1	0.34159	0.4665	1	1	0.07752
Glycine, serine and threonine metabolism	32	0.43537	1	0.36002	0.44368	1	1	0
Glycerophospholipid metabolism	32	0.43537	1	0.36002	0.44368	1	1	0.05824
Cysteine and methionine metabolism	41	0.55782	1	0.43723	0.35929	1	1	0.12222

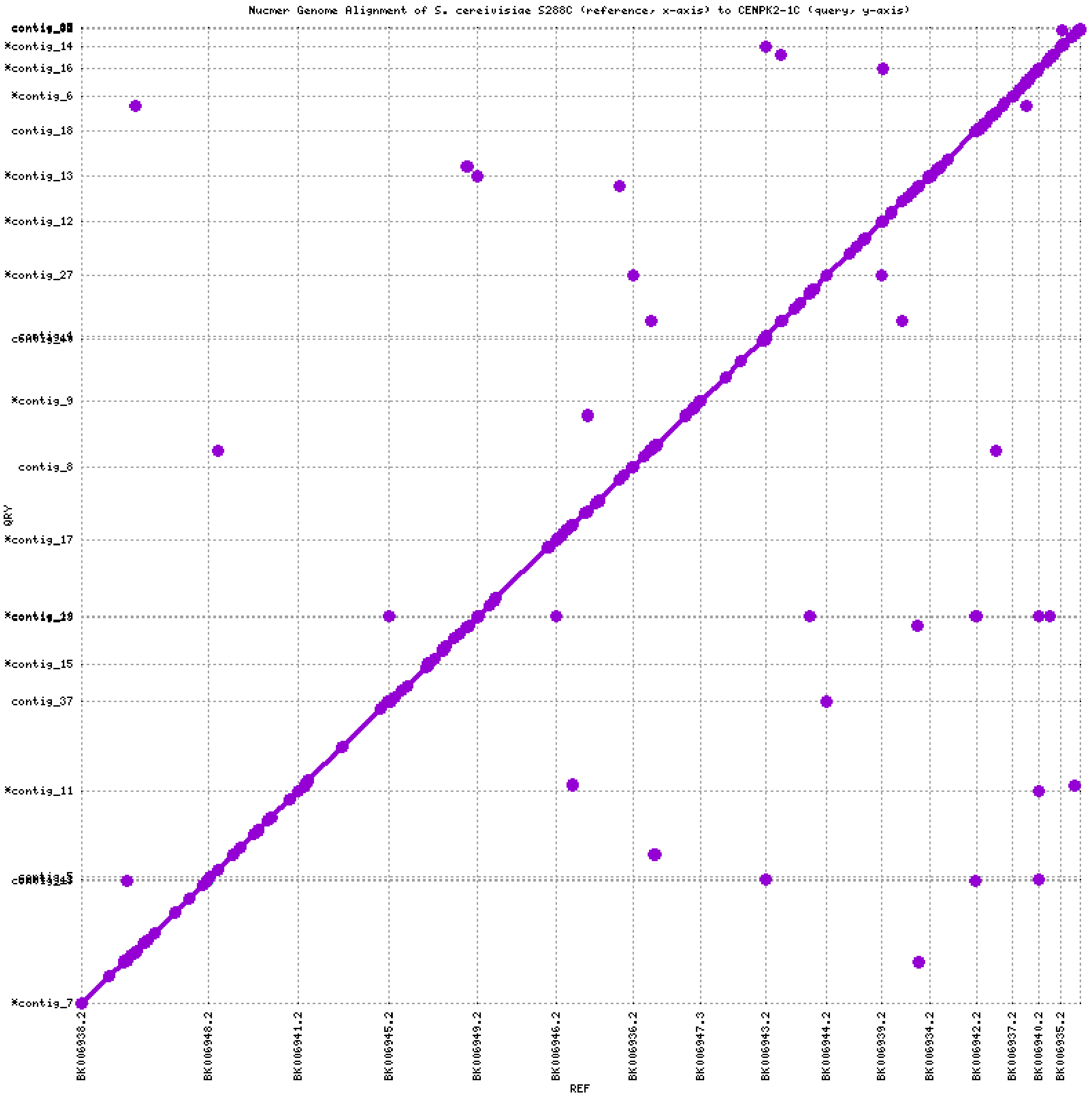

Supplementary Figure 62: Nucmer alignment of *S. cerevisiae* S288C (R64 reference) and *S. cerevisiae* CENPK2-1C. Minimum cluster length = 100, maximum gap distance = 500, filtering = yes, fat = yes. The alignment shows an overall high degree of identity with no major structural changes.

Supplementary Table 6: DNAdiff output of *S. cerevisiae* R64 S288C genome (reference) and *S. cerevisiae* CENPK2-1C (query).

	[REF]	[QRY]
[Sequences]		
TotalSeqs	28	16
AlignedSeqs	21(75.0000%)	16(100.0000%)
UnalignedSeqs	7(25.0000%)	0(0.0000%)
[Bases]		
TotalBases	11994286	12071326
AlignedBases	11886308(99.0998%)	11960630(99.0830%)
UnalignedBases	107978(0.9002%)	110696(0.9170%)
[Alignments]		
1-to-1	174	174
TotalLength	11817895	11818167
AvgLength	67918.9368	67920.5
AvgIdentity	99.7333	99.7333
M-to-M	494	494
TotalLength	12356756	12356522
AvgLength	25013.6761	25013.2024
AvgIdentity	99.6238	99.6238
[Feature	Estimates]	
Breakpoints	947	970
Relocations	11	12
Translocations	17	23
Inversions	0	0
Insertions	227	305
InsertionSum	322830	420454
InsertionAvg	1422.1586	1378.5377
TandemIns	11	4
TandemInsSum	2898	477
TandemInsAvg	263.4545	119.25
[SNPs]		
TotalSNPs	23468	23468
GT	707(3.0126%)	699(2.9785%)
GA	4370(18.6211%)	4390(18.7063%)
GC	648(2.7612%)	652(2.7783%)
TA	1044(4.4486%)	966(4.1162%)
TG	699(2.9785%)	707(3.0126%)
TC	4384(18.6808%)	4222(17.9905%)
CG	652(2.7783%)	648(2.7612%)
CT	4222(17.9905%)	4384(18.6808%)
CA	702(2.9913%)	684(2.9146%)
AT	966(4.1162%)	1044(4.4486%)
AC	684(2.9146%)	702(2.9913%)
AG	4390(18.7063%)	4370(18.6211%)
TotalGSNPs	13531	13531
GT	344(2.5423%)	354(2.6162%)
GC	333(2.4610%)	371(2.7419%)
GA	2588(19.1265%)	2635(19.4738%)
TA	547(4.0426%)	511(3.7765%)
TG	354(2.6162%)	344(2.5423%)
TC	2600(19.2151%)	2541(18.7791%)
CG	371(2.7419%)	333(2.4610%)
CT	2541(18.7791%)	2600(19.2151%)
CA	363(2.6827%)	344(2.5423%)
AT	511(3.7765%)	547(4.0426%)
AC	344(2.5423%)	363(2.6827%)
AG	2635(19.4738%)	2588(19.1265%)
TotalIndels	6524	6524
G.	424(6.4991%)	469(7.1888%)
T.	1030(15.7879%)	1398(21.4286%)
C.	369(5.6560%)	473(7.2502%)
A.	989(15.1594%)	1281(19.6352%)
N.	91(1.3948%)	0(0.0000%)
.T	1398(21.4286%)	1030(15.7879%)
.A	1281(19.6352%)	989(15.1594%)
.G	469(7.1888%)	424(6.4991%)
.C	473(7.2502%)	369(5.6560%)
.N	0(0.0000%)	91(1.3948%)
TotalGIndels	783	783
G.	27(3.4483%)	23(2.9374%)
T.	167(21.3282%)	177(22.6054%)
C.	29(3.7037%)	25(3.1928%)
A.	163(20.8174%)	172(21.9668%)
.T	177(22.6054%)	167(21.3282%)
.C	25(3.1928%)	29(3.7037%)
.G	23(2.9374%)	27(3.4483%)
.A	172(21.9668%)	163(20.8174%)

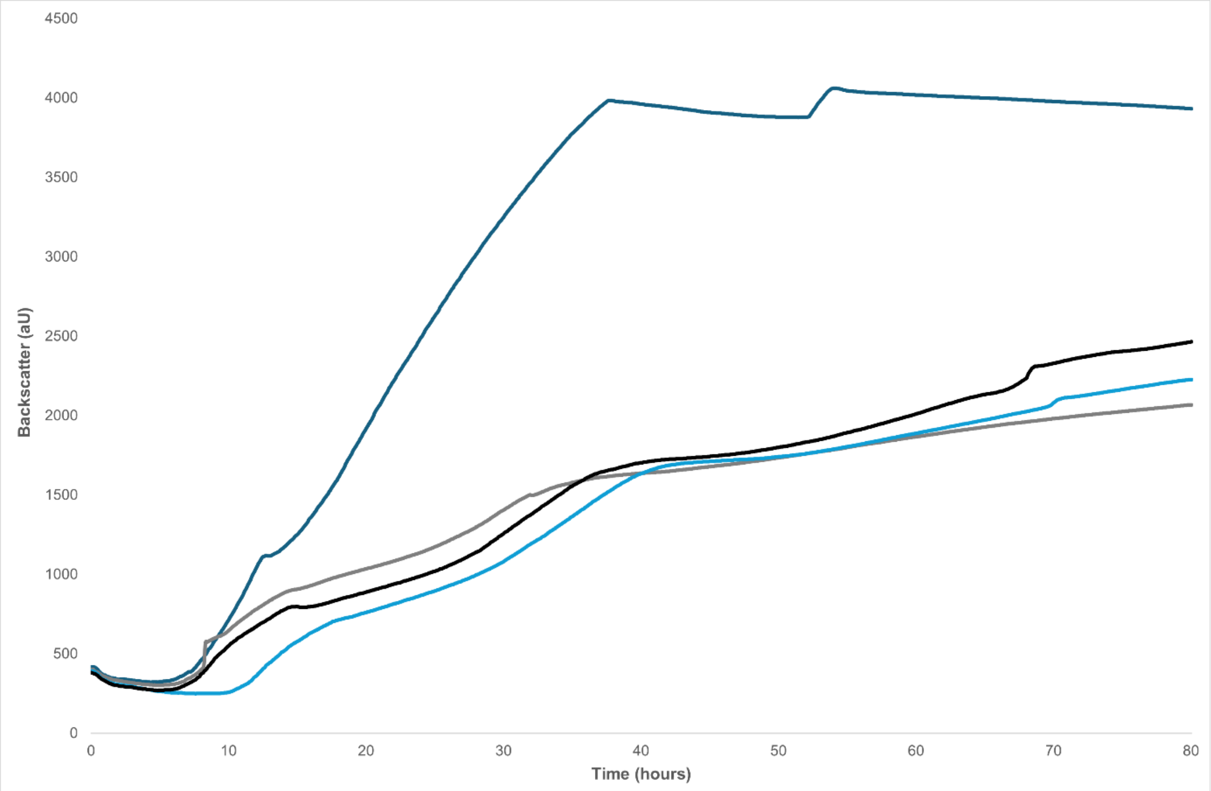

Supplementary Figure 63: Continual growth monitoring curves of Knockout-Rescue Assay, Wildtype (black), *Δpdr5::KANM* (gray), *Δpdr5::PDR5-KANM* (dark blue), *Δcis1::URA3* (light blue). The observed post-diauxic shift occurs in all strains except *Δpdr5::KANM.*

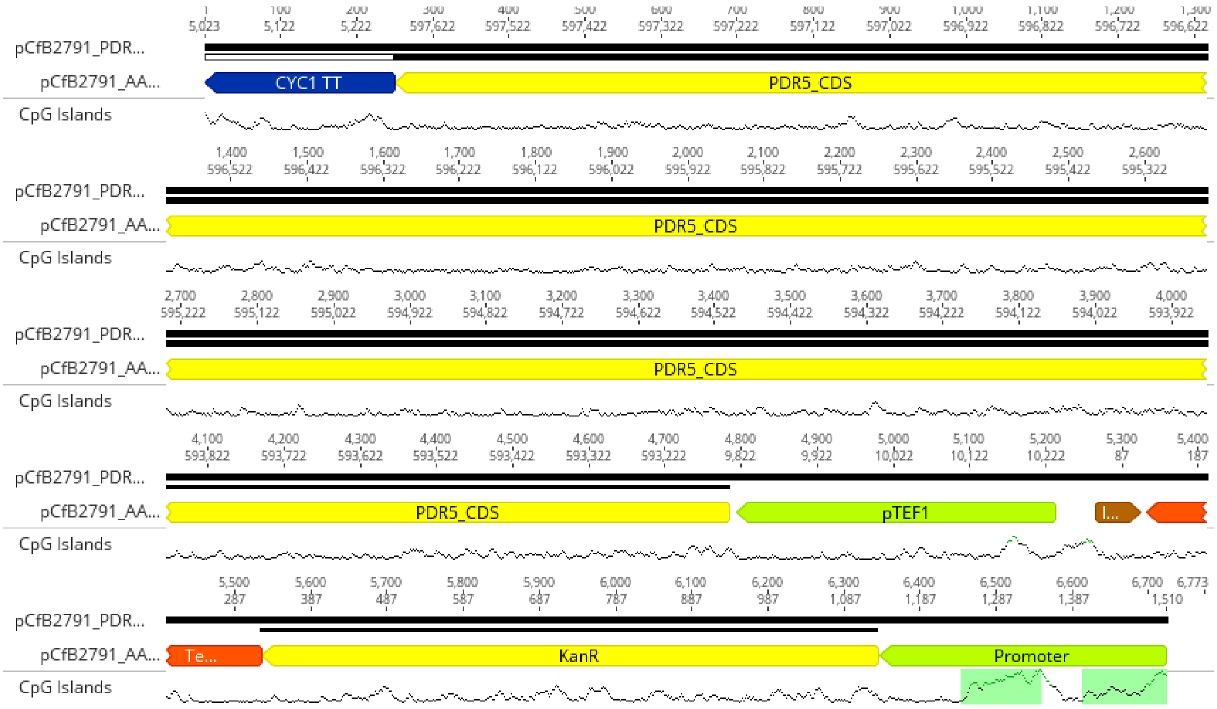

Supplementary Figure 64: Diagram of insertion cassette for Knockout-rescue assay, Δpdr5::KANM was produced with the sequence from basepairs 5,333 to 6,723. *Δpdr5::PDR5-KANM* was produced with the entire sequence.
